# Synergistic regulation of DACH1 stability by acetylation and deubiquitination promotes colorectal cancer progression

**DOI:** 10.1038/s41419-025-07696-9

**Published:** 2025-05-19

**Authors:** Liang Zhu, Can Cheng, Heng Li, Jingwen Liu, Yang Wu, Liang Wu, Hanhui Yao

**Affiliations:** 1 Department of Gastric Surgery, The First Affiliated Hospital of USTC, Division of Life Sciences and Medicine, University of Science and Technology of China, Anhui Province Key Laboratory of Hepatopancreatobiliary Surgery, Hefei, China; 2 Department of Vascular Surgery, The First Affiliated Hospital of USTC, Division of Life Sciences and Medicine, University of Science and Technology of China, Anhui Province Key Laboratory of Hepatopancreatobiliary Surgery, Hefei, China; 3 Department of Comprehensive Surgery, Anhui Provincial Cancer Hospital, West District of The First Affiliated Hospital of USTC, Division of Life Sciences and Medicine, University of Science and Technology of China, Heifei, China; 4 Health Management Center, The First Affiliated Hospital of USTC, Division of Life Sciences and Medicine, University of Science and Technology of China, Heifei, China

**Keywords:** Colorectal cancer, Oncogenesis

## Abstract

Colorectal cancer (CRC) remains a significant challenge in oncology, with limited therapeutic options for aggressive subtypes. This study elucidates the critical roles of USP7 and DACH1 in CRC, revealing their involvement in tumor progression and potential as therapeutic targets. USP7 was identified as a deubiquitinase that stabilizes DACH1, enhancing its tumor-promoting activities. Mechanistically, USP7 directly interacts with DACH1, protecting DACH1 from UHRF1-induced ubiquitination and degradation by removing ubiquitin chains, particularly K48-linked types, which are crucial for protein degradation. Additionally, acetylation at K680 on DACH1, mediated by acetyltransferase GCN5, enhances its interaction with USP7, further influencing DACH1 stability and function. Clinically, high levels of USP7 and DACH1 correlate with poor prognosis in CRC patients, underscoring their significance in disease progression. These findings suggest that targeting the USP7-DACH1 axis could offer a novel therapeutic strategy for managing CRC, particularly in forms characterized by aggressive and metastatic behaviors.

## Introduction

Colorectal cancer (CRC), a malignancy arising from epithelial cells in the colon or rectum, is a significant contributor to cancer-related mortality globally [1, 2]. Post-translational modifications, specifically ubiquitination and deubiquitination, are essential for controlling tumor growth and spread [3–6]. Ubiquitination typically marks proteins for degradation, whereas deubiquitination stabilizes proteins by removing these marks, thereby influencing cellular activities. Dysregulation of this balance can lead to the development and spread of tumors. Therefore, a deeper understanding of these processes is essential to identify potential therapeutic targets.

DACH1 is indeed well-established as a chromatin-binding protein. It that associates with other DNA-binding transcription factors to regulate gene expression and cell fate determination during development [7]. DACH1 has been shown to associate with promoter regions of target genes, influencing chromatin structure and transcriptional outcomes [8, 9]. The role of DACH1 in cancer is multifaceted and varies depending on the specific cancer type. While DACH1 usually functions as a tumour suppressor in renal cancer [10], lung adenocarcinoma [11], prostate cancer [12] and breast cancer [8], inhibiting biological processes such as cell growth or promoting apoptosis, mainly through the inhibition of cell-cycle proteins, its role in CRC seems to be different [13]. Recent studies using organoid models have indicated that DACH1 may act as a tumor promoter in CRC by modulating BMP signaling pathways [13]. The upregulation of DACH1 in CRC is strongly correlated with increased tumor proliferation, invasion, and metastasis, suggesting that DACH1 may possess distinct biological functions and mechanisms within this particular cancer subtype. This contradictory observation has heightened the interest in elucidating the role of DACH1 in CRC.

Deubiquitinating enzyme USP7, also known as HAUSP, influences tumor survival and progression by stabilizing key proteins in various cancers [14, 15]. For instance, in neuroblastoma, USP7 facilitates the growth and survival of cancer cells by deubiquitinating and stabilizing N-myc [16, 17]. In nasopharyngeal carcinoma, USP7 enhances tumor invasiveness and chemotherapy resistance by stabilizing KDM5B [18]. Moreover, the inhibition of USP7 has shown potential in triple-negative breast cancer by destabilizing FOXM1, thus suppressing tumor growth and underscoring its significance as a therapeutic target in cancer treatment [19]. In this study, the function of USP7 in CRC was found to be critical. USP7 effectively protects DACH1 from UHRF1-induced ubiquitination and degradation, thereby stabilizing DACH1 protein. This mechanism highlights the integral role of USP7 in modulating protein stability, which is essential for cancer cell viability and progression.

Deubiquitination and acetylation play crucial roles in regulating protein stability, activity, and interactions within the cell [20, 21]. Acetylation directly affects the function of deubiquitinases and modifies the stability and activity of their substrate proteins. This dual mechanism underscores the complexity and breadth of acetylation in regulating protein fate and cellular signaling, highlighting its potential key role in both physiological and pathological processes. For example, acetyltransferase GCN5 has been identified as a factor that increases the binding affinity between USP1 and PARP1 by acetylating specific sites, thereby contributing to the stabilization of PARP1 [22]. Furthermore, acetylation of the MCL1 protein enhances its interaction with the deubiquitinating enzyme USP9X, leading to deubiquitination and subsequent stabilisation, which plays a crucial role in promoting tumour cell survival and pathological development [23]. Our research focused on investigating the acetylation modifications of the DACH1 protein and its implications for tumor biology. Our study revealed that the acetyltransferase GCN5 mediates the acetylation of DACH1 at K680, leading to a notable improvement in the interaction between DACH1 and the deubiquitinase USP7, consequently improving the stability of DACH1. This interplay between acetylation and deubiquitination is crucial in modulating the functionality of DACH1, shedding light on the underlying mechanisms of CRC, and presenting potential molecular targets for novel therapeutic interventions.

## Materials and methods

### Plasmid transfection and lentiviral infection

In this study, the following plasmids were constructed using pcDNA3.1 (37680, Addgene): Myc-DACH1-FL, Myc-DACH1-N, Myc-DACH1-SKI/SNO/DAC domain, Myc-DACH1-M1 domain, Myc-DACH1-M2 domain, Myc-DACH1-DACHbox-C, HA-USP7-FL, HA-USP7-TRAF like domain, HA-USP7-Catalytic domain, HA-USP7_ICP0-bdg, HA-USP7_C2, Myc-DACH1, HA-USP7, and HA-USP7_C223A, Flag-UHRF1, along with His-ubiquitin (His-Ubi) and its lysine-specific mutants: His-K6, His-K11, His-K27, His-K29, His-K33, His-K48, His-K63, Flag-PCAF, Flag-p300, Flag-GCN5, Flag-Tip60, Flag-CBP, Myc-DACH1-K680Q, and Myc-DACH1-K680R. GST-USP7 plasmids were generated using pGEX-4T-1 (129567, Addgene). Lentiviral plasmids encoding Myc-DACH1, HA-USP7, Sh-USP7, and Sh-DACH1 were constructed and introduced into CRC cells via viral transduction. The sequences for Sh-USP7 and Sh-DACH1 are provided in Table S1. Infected cells were supplemented with polybrene (HY-112735, MedChemExpress) and stable cell lines were established through puromycin selection.

### Antibodies and other reagents

Cell Signaling Technology (CST, Danvers, MA, USA) supplied the anti-Myc (#2276), anti-HA (#3724), anti-DYKDDDDK (#14793), and anti-His (#12698) antibodies. Proteintech (Wuhan, China) provided anti-USP7 (#66514-1-Ig), anti-DACH1 (#10914-1-AP), anti-Histone-H3 (#17168-1-AP), anti-UHRF1 (#21402-1-AP), and anti-GAPDH (#10494-1-AP) antibodies. PTM BioLab (Hangzhou, Zhejiang, China) supplied the anti-Ub (#PTM-1124RM, PTM-5798) and anti-acetyllysine antibodies (#PTM-105RM). MedchemExpress (MCE) in New Jersey, USA provided P5091 inhibitor (#HY-15667), anti-HA (#HY-K0201), anti-c-Myc (#HY-K0206A) and anti-His (#HY-K0209) agarose beads. SelleckChem (Houston, Texas, USA) supplied the MG132 (#S2619) and CQ (#S6999).

### Immunofluorescence

Immunofluorescence was conducted on a confocal microscope slide according to the manufacturer’s guidelines. Cells were prepared and stained with specific antibodies and fluorescence-dye-conjugated secondary antibodies, with DAPI used for nuclear staining.

### Deubiquitination of DACH1 in vivo and in vitro

In this study, we investigated DACH1 ubiquitination in various cell lines treated with MG132. Cell lysis was performed using RIPA buffer and protease inhibitors, followed by incubation with specific antibodies. Myc-DACH1 and His-Ubi were transfected into HEK293T cells for in vitro deubiquitination assay. DACH1 was isolated using anti-Myc beads at 48 h post-transfection. HA-USP7 and USP7_C223A were expressed and purified using HEK293T cells. The c-Myc binding beads were washed three times with PBS and then analysed by IB. In the Ni-NTA pull-down assay, cells lysed in RIPA buffer containing protease inhibitors were exposed to Ni-NTA resin for 1 h at 4 °C and then washed three times with binding buffer.

### Immunohistochemistry (IHC)

Tissue microarrays (TMAs) containing paired cancerous and normal tissues were obtained from Shanghai Outdo Biotech Co., Ltd. IHC staining was performed on paraffin-embedded tissue blocks of DACH1 and USP7 cells. The staining intensity and proportion were then assessed in a semi-quantitative manner, with intensity graded on a scale of 0–3 and proportion on a scale of 1–4. IHC staining was performed by two independent pathologists, and the score for USP7 or DACH1 was determined by the product of the intensity and extent of staining. an H-score ≥6 indicated high expression, whereas an H-score <6 indicated low expression.

### Animal model

BALB/c nude mice were acquired from Beijing Vital River Laboratory Animal Technology Co., Ltd (Beijing, China). In vitro cultured CRC cell lines were dissociated using 0.25% trypsin-EDTA upon reaching approximately 80% confluency. Following dissociation, the cells were washed and resuspended in PBS to a density of 1 × 10^7^ cells/ml. To establish a subcutaneous xenograft model, 300 µL of the cell mixture was injected into the right flank of each mouse. The development of xenografts was monitored starting one week post-injection, with caliper measurements taken three times per week, using the formula 1/2 × L × W × W (where W the shortest diameter and L represents the longest diameter) to calculate tumor volume. In experiments involving P5091, one week post-injection, animals were administered intraperitoneally with P5091 (15 mg/kg) or vehicle three times per week for two consecutive weeks. Subsequently, the mice were euthanized in a humane manner and the xenografts were removed and weighed. Metastasis was evaluated by euthanizing the mice six weeks after injection and subjecting their lungs to histological analysis.

### Statistical data

Comparisons between groups were performed using t-test, correlation between USP7 and DACH1 using chi-square test, and Kaplan-Meier survival curve using log-rank test. Statistical significance was set at p ≤ 0.05. Quantitative analyses were performed using GraphPad Prism 8 or Microsoft Excel 2019 software. Results from three independent experiments were consistent unless otherwise stated in the figure legends.

## Results

### The elevated protein levels of DACH1 in CRC were correlated with higher tumor malignancy and poorer patient prognosis

**Fig. 1:**
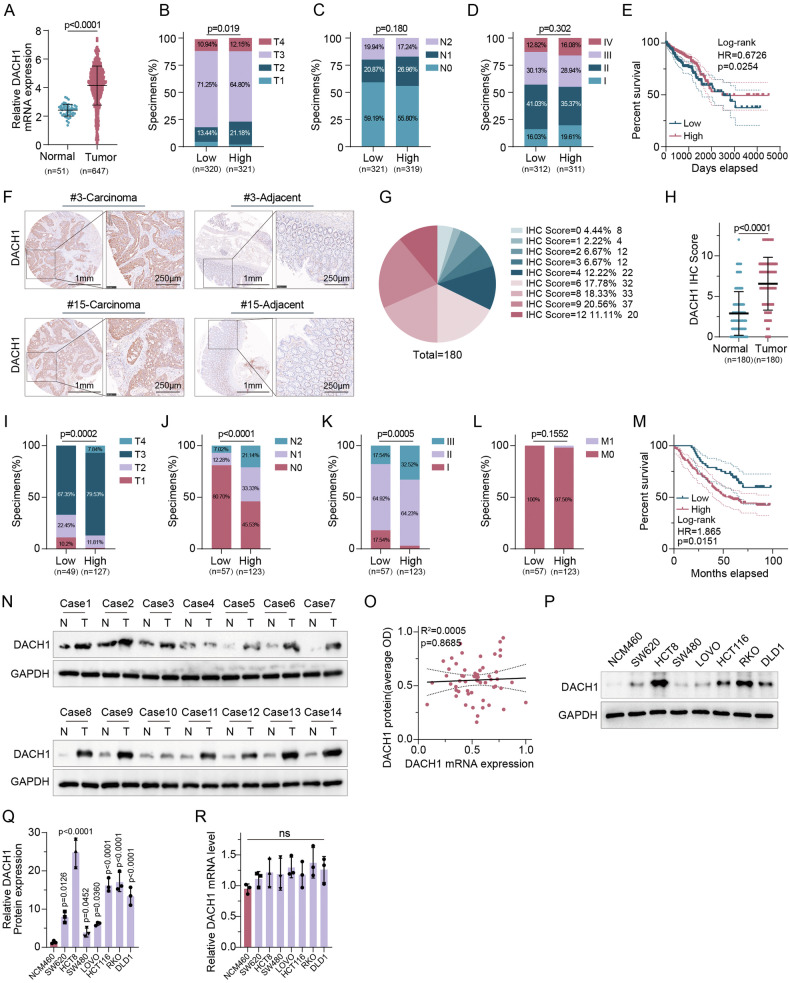
The elevated protein levels of DACH1 in CRC were correlated with higher tumor malignancy and poorer patient prognosis. **A** Analysis of DACH1 mRNA expression was performed in the TCGA CRC dataset. An unpaired t-test was utilized to compare cancerous tissues with adjacent normal tissues or matched adjacent tissues. Correlation between DACH1 mRNA expression and tumour T-stage (**B**), N-stage (**C**), and pathological stage (**D**) in the TCGA CRC dataset. χ2 test (two-sided). **E** Kaplan–Meier analysis of TCGA CRC dataset stratified by low and high levels of DACH1 mRNA (log-rank test, n = 644). **F** Representative image of DACH1 IHC staining in a human CRC sample. Scale bar, 250 µm. **G** Percentage of samples with high, medium, and low IHC staining scores. **H** The IHC score of DACH1 in CRC and matched adjacent tissues. Comparison between cancerous tissues and adjacent normal tissues can be conducted using the Wilcoxon matched-pairs test. Correlation between DACH1 protein expression and tumour T-stage (**I**), N-stage (**J**), pathological stage (**K**) and M-stage (**L**) in 180 human CRC specimens. χ2 test (two-sided). **M** Kaplan–Meier analysis for CRC patients stratified by high versus low levels of DACH1 protein (log-rank test, *P* = 0.0151, *n* = 180). **N**, **O** IB analysis was performed on cell lysates from 56 pairs of CRC tumor tissues and matched adjacent tissues to detect the expression of DACH1 (**N**). The Spearman correlation analysis (two-sided) revealed a weak correlation between DACH1 protein levels and DACH1 mRNA expression (**O**), R^2^ = 0.0005, P = 0.8685. **P**–**R** IB and qRT-PCR analyses of DACH1 protein in the indicated cells. In Q and R, error bars represent the mean ± SD of three independent experiments, ns not significant. One-way repeated-measures ANOVA test. Source data are provided as source data files.

Initially, we investigated the expression patterns of DACH1 in CRC and examined its potential link with disease progression and outcomes. Analysis of DACH1 mRNA levels showed upregulation in tumor tissues compared to normal tissues and a statistical association with T stage (p = 0.019), as depicted in Fig. 1A and Fig. 1B. The results showed that there was no significant correlation between DACH1 mRNA expression levels and lymph node metastatic status (stage N), distant metastasis (stage M), or pathological stage, as shown in Figs. 1C, S1A, D. However, DACH1 mRNA expression levels did not distinguish between different T stages (Fig. S1B). Additionally, the analysis revealed that high DACH1 mRNA expression did not significantly correlate with the overall survival rates of patients, suggesting that mRNA levels alone may not accurately reflect the association between DACH1 expression and tumor progression or prognosis (Fig. 1E).

A different picture emerged when DACH1 protein levels were analyzed using IHC. Compared to adjacent normal tissues, a significant increase in DACH1 protein expression was observed in CRC tissues (Fig. 1F–H). Importantly, higher DACH1 protein levels were closely associated with higher T, N, and pathological stages, indicating that elevated DACH1 protein expression may be linked to increased aggressiveness and metastatic capability of the tumors (Fig. 1I–K). With a restricted sample size of cases exhibiting distant metastasis, no substantial correlation was found between elevated DACH1 protein expression and M stage. However, notable variations in DACH1 protein expression were observed among the various T and N stages, demonstrating a gradual increase in expression levels with advancing stages. These findings underscore the potential of DACH1 as a biomarker for tumor progression and prognosis.

This study delved deeper into the association between DACH1 mRNA expression and protein levels. The results revealed a lack of substantial correlation between the expression of DACH1 mRNA and protein, with fluctuations in DACH1 protein levels not corresponding to alterations in mRNA levels among the various cell lines (Fig. 1N–R). These observations indicate that protein levels of DACH1, as opposed to mRNA levels, play a more significant role in CRC progression and patient outcomes, suggesting that the regulation of DACH1 expression may primarily occur post-translationally. Overall, these results underscore the close association between DACH1 protein expression and tumor proliferation, invasiveness, and patient prognosis in CRC, while DACH1 mRNA levels did not accurately reflect this link. This highlights the importance of assessing DACH1 protein levels for predicting the pathological characteristics and prognosis of CRC patients and underscores the necessity of studying the post-translational regulation of DACH1 protein in tumors.

### USP7 interacts with DACH1

**Fig. 2:**
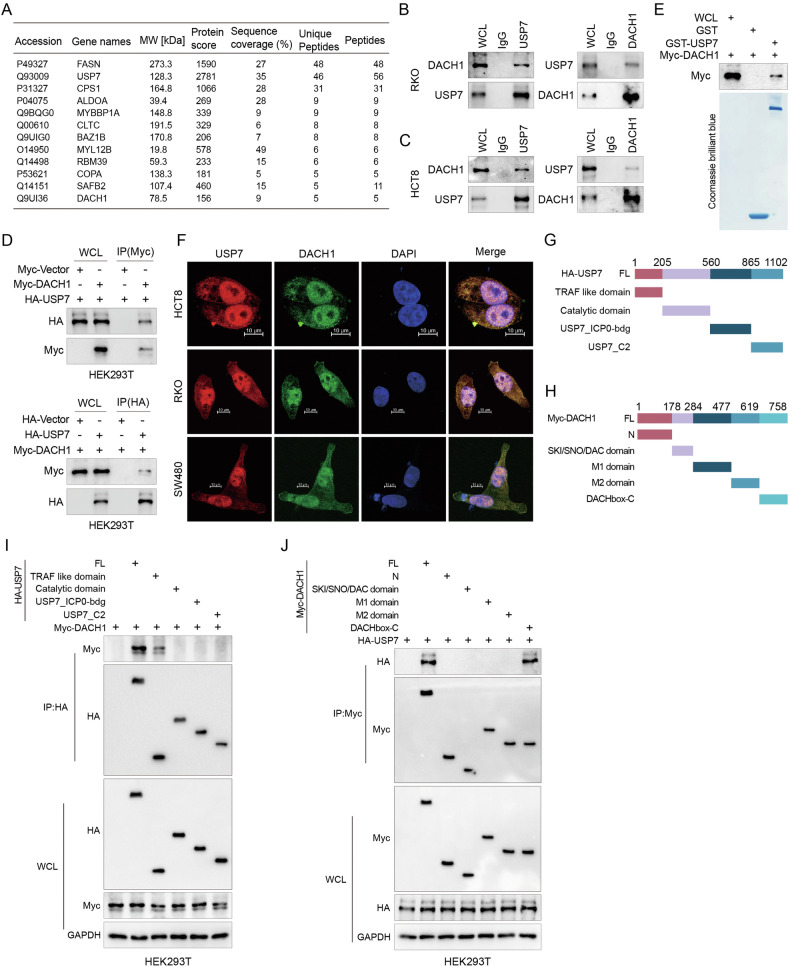
USP7 interacts with DACH1. **A** Myc-DACH1 was transfected into HCT8 cells and immunoprecipitated with anti-Myc antibody. Mass spectrometry analysis was then performed. **B**, **C** RKO and HCT8 cells were immunoprecipitated with anti-DACH1 or anti- USP7 and analysed. **D** HEK293T cells were co-transfected with HA-USP7 and Myc-DACH1. Cell lysates were then subjected to IP detection using anti-HA or anti-Myc antibodies. **E** Purified DACH1 was incubated with GST or GST-USP7 conjugated with GSH-Sepharose followed by Coomassie blue staining. **F** Representative images after immunofluorescence staining of fixed HCT8, RKO and SW480 cells with DACH1 (green) and USP7 (red) antibodies. Cell nuclei were counterstained with DAPI (blue). Scale bar is 10 μm. **G**, **H** Schematic representation of HA-tagged full-length (FL) USP7, Myc-tagged FL DACH1 and their various deletion mutants. **I** Myc-DACH1 and HA-tagged FL USP7 or its deletion mutants were co-transfected in HEK293T cells and analysed by IP using HA or Myc magnetic beads, followed by IB analysis using Myc and HA antibodies. **J** In HEK293T cells, HA-USP7 was cotransfected with Myc-tagged FL DACH1 or its deletion mutants and subjected to IP analysis using Myc magnetic beads followed by IB analysis using antibodies against HA and Myc.

HCT8 cells stably expressed the Myc-tagged DACH1, and whole cell extracts were subsequently purified using anti-Myc affinity beads and subjected to mass spectrometry to identify proteins that may interact with DACH1. The results depicted in Fig. 2A and Table S3 indicate that USP7 displayed significant peptide coverage in DACH1 immunoprecipitates, suggesting a potential binding relationship between USP7 and DACH1. The initial screening of USP7, identified from mass spectrometry data, was further supported by predictions from the UbiBrowser software (http://ubibrowser.ncpsb.org/ubibrowser/) targeting DACH1 as a deubiquitinating enzyme (Fig. S2A). Further exploration of USP7 and DACH1 interactions in vivo was conducted using co-immunoprecipitation (Co-IP) experiments. In various cell lines, including RKO, HCT8, and SW480, endogenous USP7 interacted with endogenous DACH1 (Figs. 2B, C, and S2B). Co-precipitation of HA-tagged USP7 and Myc-tagged DACH1 was observed in both HEK293T (Fig. 2D). To verify whether this interaction was direct, GST pull-down assay was performed. Purified GST-tagged USP7 bound directly to Myc-tagged DACH1, demonstrating significantly enhanced binding compared to the GST, further confirming the direct interaction between USP7 and DACH1 (Fig. 2E). Immunofluorescence staining revealed that endogenous DACH1 and USP7 primarily colocalized in the nucleus of CRC cells (Fig. 2F), suggesting a potential joint participation in nuclear biological processes. Finally, truncation mutation analysis of DACH1 and USP7 indicated that the TRAF-like domain of USP7 and DACHbox-C domain of DACH1 are essential for their binding (Fig. 2G–J). These findings provided a more specific structural basis for the interaction between DACH1 and USP7. Collectively, our results revealed a specific direct interaction between USP7 and DACH1, providing a molecular foundation for further exploration of DACH1’s function in CRC and its potential as a therapeutic target.

### USP7 maintains DACH1 stability

**Fig. 3:**
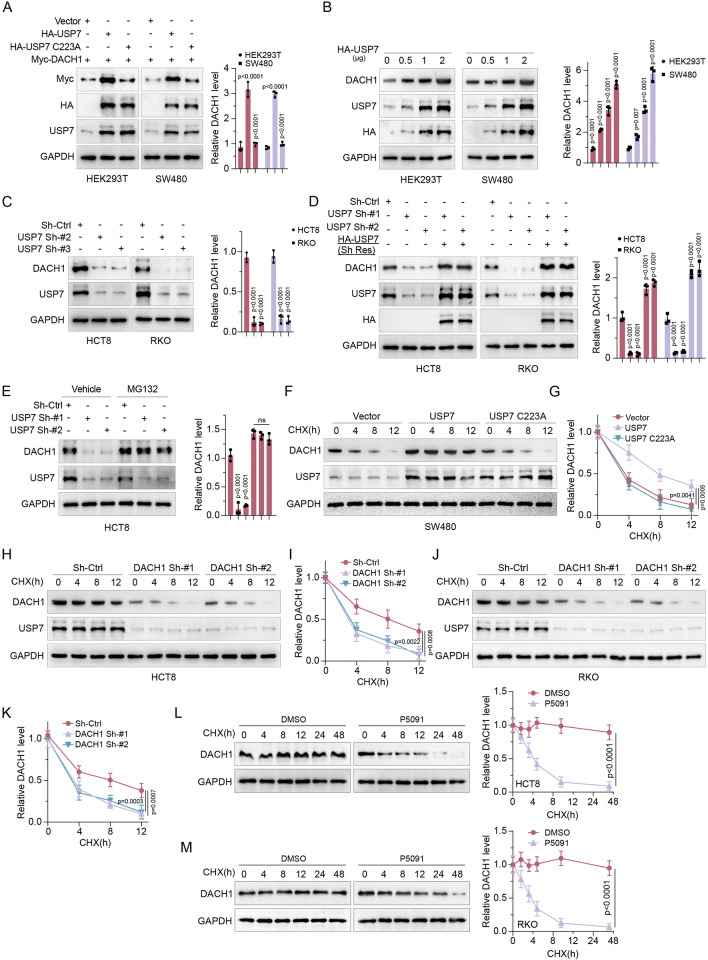
USP7 maintains DACH1 stability. **A** IB analysis of the indicated cells transfected with Myc-DACH1 and USP7 plasmids or USP7_C223A for 48 h. **B** IB analysis of the indicated cells transfected with USP7 plasmids for 48 h. **C** IB analysis of the indicated cells transfected with shRNA-Ctrl (Control) and USP7 shRNAs. **D** IB analysis of cells transfected with USP7 shRNAs and shRNAs-resistant (Sh-Res) HA-USP7. **E** HCT8 cells transfected with two independent USP7 shRNAss were treated with or without the proteasome inhibitor MG132 (20 μM, 8 h) and then analysed for USP7 and DACH1. **F**, **G** SW480 cells were stably transfected with USP7 or USP7_C223A, then treated with CHX (50 μg/ml) for the indicated times and lysates were analysed. Graphs show the amount of DACH1 protein remaining after CHX treatment as a percentage of the starting DACH1 protein level. **H**–**K** HCT8 and RKO cells stably transfected with shRNA-Ctrl and USP7 shRNAs were treated with CHX (50 μg/ml) for the indicated times and lysates were analysed. Graphs show the amount of DACH1 protein remaining after CHX treatment as a percentage of the starting DACH1 protein level. **L**, **M** The indicated cells were treated with P5091 (12.5 μM) or DMSO and lysates were analysed. Error bars represent the mean ± SD of three independent experiments (**A**–**M**). In **L**, **M**, two-sided Student’s *t*-test. In **A**–**K**, One-way repeated-measures ANOVA test.

To assess the influence of USP7 on the stability of DACH1 in CRC cells, the expression of DACH1 and its half-life were analyzed. The findings demonstrated that upregulation of USP7 led to a significant increase in DACH1 levels (Fig. 3A) in a dose-dependent manner (Fig. 3B), whereas the use of specific shRNAs targeting USP7 resulted in a notable decrease in endogenous DACH1 levels (Figs. 3C and S3A). To investigate whether the enhanced DACH1 protein levels were attributable to the deubiquitinase activity of USP7, both wild-type (WT) and mutant form (USP7_C223A) of USP7 were introduced into SW480 and HEK293T cells. The findings of this study indicated that only the presence of USP7-WT resulted in an increase in DACH1 levels, while USP7_C223A did not affect protein abundance (Fig. 3A). Conversely, USP7 did not influence DACH1 mRNA expression (Fig. S3B, C). Additionally, co-expression of USP7 shRNAs with a USP7 shRNAs-resistant overexpression plasmid in HCT8 and RKO cells led to an augmentation in endogenous DACH1 levels (Fig. 3D). Further investigation of the role of USP7 in DACH1 protein stability revealed that depletion of USP7 resulted in a reduction in DACH1 expression, which was largely restored by the addition of MG132 (Fig. 3E). Overexpression of USP7 WT but not the catalytically inactive mutant USP7_C223A resulted in a prolonged half-life and increased DACH1 levels (Fig. 3F, G). Conversely, USP7 knockdown led to a shortened half-life and decreased levels of DACH1 (Fig. 3H–K). Additionally, treatment with the selective small molecule inhibitor of USP7, P5091, significantly reduced the half-life of the DACH1 protein, as illustrated in Fig. 3L, M. These findings suggest that USP7 specifically modulates the stability of DACH1 in an enzymatic activity-dependent manner without affecting DACH1 mRNA levels.

### USP7 deubiquitinates DACH1

**Fig. 4:**
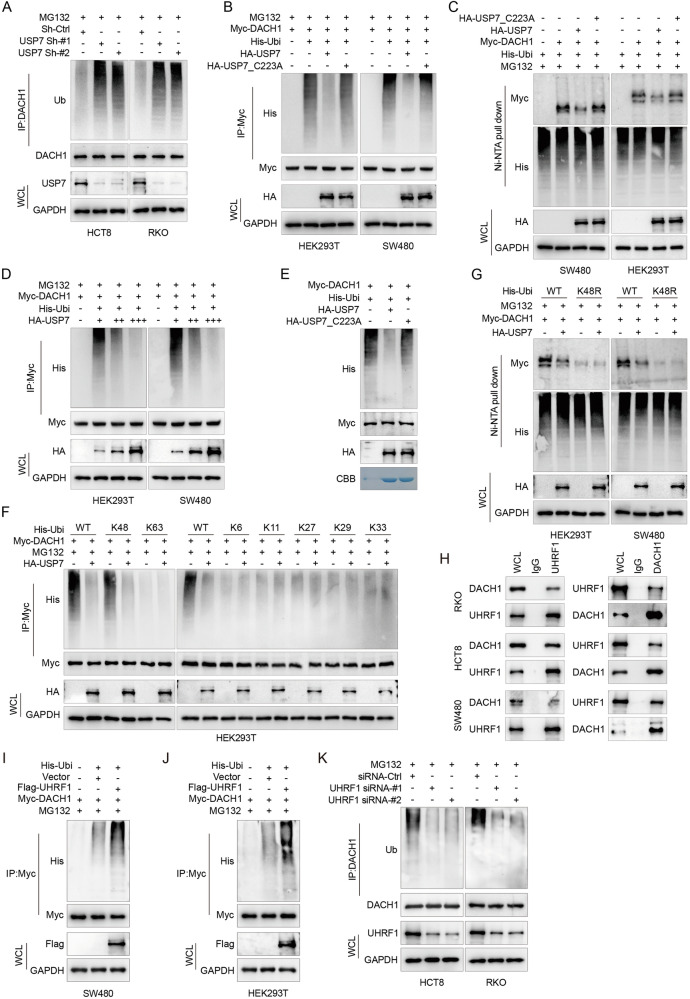
USP7 deubiquitinates DACH1. **A** HCT8 and RKO cells stably transfected with shRNA-Ctrl and USP7shRNAs were harvested by treatment with MG132 (10 µм) for 6 h. Lysates were immunoprecipitated with anti-DACH1 and analysed. **B** HEK293T or SW480 cells were co-transfected with Myc-DACH1, His-Ubi and HA-USP7 WT or USP7_C223A. Cell lysates were IP with Myc beads followed by IB with anti-His and anti-Myc antibodies. Cells were treated with 20 μM MG132 for 8 h and harvested. **C** After co-transfection of cells with the indicated plasmids, His-Ubi was pulled up with Ni-NTA beads, followed by IB to detect polyubiquitinated DACH1 protein. **D** Increasing amounts of USP7 were transfected into HEK293T and SW480 cells, which were treated with MG132 for 8 h before collection. Cell lysates were IP with Myc beads followed by IB with anti-His and anti-Myc antibodies. **E** In vitro deubiquitination assay was performed. Ubiquitinated Myc-DACH1 proteins were treated with USP7 WT or USP7_C223A. Myc-DACH1 was immunoprecipitated with Myc beads, followed by IB with anti-HA and anti-His antibodies. recombinant USP7 WT or USP7_C223A was analysed by SDS-PAGE and Coomassie Brilliant Blue staining. **F** HEK293T cells were co-transfected with Myc-DACH1, HA-USP7 and His-Ubi WT, K6 (especially K6 only), K11, K27, K29, K33, K48 or K63 plasmids to analyse ubiquitination linkage of DACH1. **G** HEK293T cells were cotransfected with Myc-DACH1, HA-USP7 and His-Ubi WT or K48R (specifically only K48 mutated to Arg). His-Ubi was pulled down with Ni-NTA beads to analyse the ubiquitination linkage of DACH1. **H** RKO, HCT8, and SW480 cells were immunoprecipitated with anti-DACH1 or anti-UHRF1 and analyzed. **I**, **J** His-Ubiquitin (Ubi) was co-transfected with Flag-UHRF1 into SW480 (**H**) or HEK-293T (**I**) cells. Cells were treated with MG132 (20 µM) for 6 h before being harvested, followed by IP with anti-Myc and analysis. **K** Myc-DACH1 was transfected into HCT8 and RKO cells that stably express control siRNA or UHRF1 siRNA. Cells were treated with MG132 (20 µM) for 6 h before being harvested, followed by IP with anti-Myc and analysis.

To investigate whether USP7 catalyzes the deubiquitination of DACH1, deubiquitination assays were conducted under various conditions. USP7 knockdown increased ubiquitination of DACH1 in HCT8 and RKO cells (Fig. 4A). Myc-DACH1 and His-Ubi were co-transfected with either WT or C223A mutant USP7 into HEK293T and SW480 cells to study the mediation of DACH1 deubiquitination by USP7. The findings of this study demonstrated that USP7-WT significantly decreased the ubiquitination of DACH1, while USP7_C223A did not exhibit any effect, as illustrated in Fig. 4B, C. Moreover, the dose-dependent relationship between USP7-WT and ubiquitination of DACH1 in HEK293T cells is shown in Fig. 4D. Additionally, in vitro deubiquitination assays confirmed the specificity of USP7 for removing ubiquitin chains from DACH1, with the USP7_C223A mutant showing no such activity (Fig. 4E). The proteasomal degradation of proteins is commonly initiated by Lys48-linked ubiquitin chains, whereas Lys63-linked chains are predominantly associated with non-proteasomal degradation pathways. To determine the precise ubiquitin chains targeted by USP7 on DACH1, all seven lysine-specific ubiquitin mutants were used. The results indicated that USP7 specifically eliminated ubiquitin chains linked to K48 on DACH1 under different experimental conditions (Fig. 4F, G, and S4A). As a ubiquitin-specific protease, USP7 deubiquitinates and stabilizes the DACH1 protein. In fact, mass spectrometry analysis of HCT8 cells identified several putative E3 ligases, including E3 ubiquitin-protein ligase UHRF1. Based on the subcellular localization and biological function of these proteins, we first confirmed the interaction between DACH1 and UHRF1 through Co-IP (Fig. 4G, and S4B). Additionally, the overexpression of UHRF1 significantly increased the ubiquitination of DACH1 (Fig. 4H, I). Conversely, UHRF1 knockdown abolished DACH1 ubiquitination after MG132 treatment (Fig. 4J). In vitro ubiquitination assays further confirmed that UHRF1 directly ubiquitinates DACH1 (Fig. S4C). We also demonstrated that UHRF1 ubiquitinates DACH1 through K48-linked ubiquitin chains (Fig. S4D, E). These data indicate that UHRF1 is a direct E3 ligase for DACH1. Next, we tested the ability of USP7 to protect DACH1 from UHRF1-mediated degradation. The overexpression of UHRF1 reduced DACH1 levels, while USP7 overexpression rescued DACH1 levels in cells overexpressing UHRF1; however, USP7_C223A, the USP7 mutant, failed to rescue DACH1 levels in UHRF1-overexpressing cells (Fig. S4F, G). Moreover, USP7 overexpression but not USP7_C223A significantly decreased DACH1 ubiquitination in UHRF1-overexpressing cells (Fig. S4H, I). These results demonstrate that USP7 effectively protects DACH1 from UHRF1-induced ubiquitination and degradation.

### USP7 promotes DACH1-mediated proliferation and metastasis in CRC

**Fig. 5:**
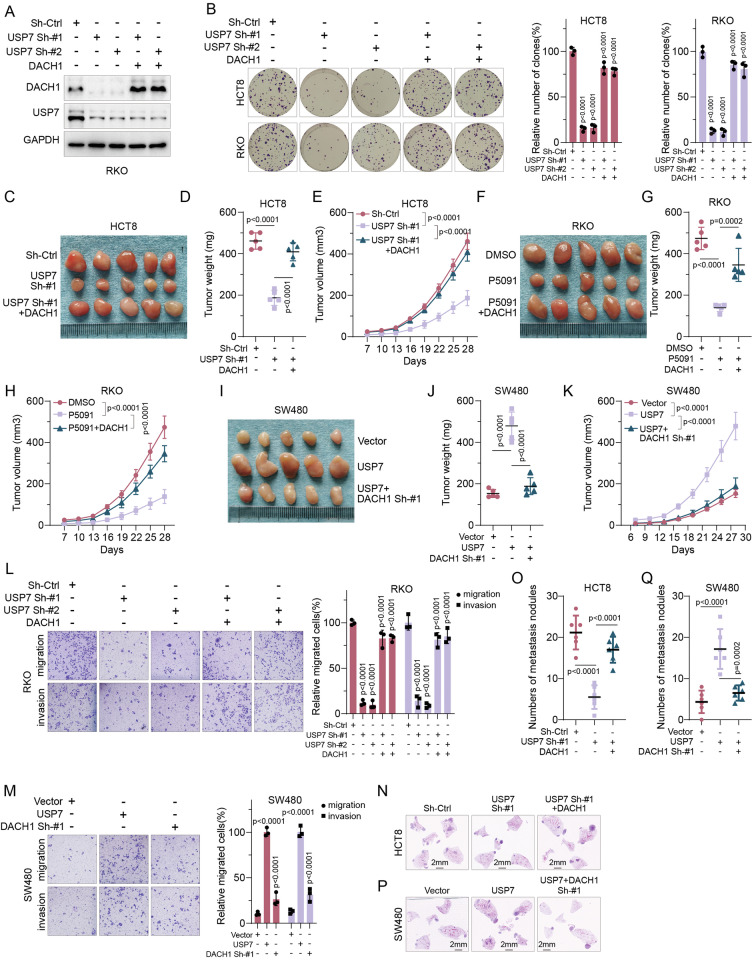
USP7 promotes DACH1-mediated proliferation and metastasis in CRC. **A** IB analysis of RKO cells transduced with shRNA-Ctrl and USP7 shRNAs with vector or DACH1 plasmid. **B** Clone formation assay of said cells. **C**–**E** Xenograft tumour assays using said cells and quantitative statistics on tumour weight (**D**) and dynamic volume (**E**). **F**–**H** Xenograft tumour formation and quantitative statistics of tumour weight (**G**) and dynamic volume (**H**) of RKO cells in response to P5091 pharmacology. **I**–**K** Xenograft tumour formation of SW480 cells stably overexpressing USP7 co-transfected with shRNA-Ctrl or DACH1-specific shRNAs and quantitative statistics of xenograft tumour weight (**J**) and dynamic volume (**K**). **L** Transwell assays were performed on the cells shown in Panel A to assess the invasive and metastatic ability of the cells and quantitative statistics were performed. **M** Transwell assays were performed on the cells shown in Panel I to assess cell invasion and metastatic ability and quantitative statistics were performed. **N**–**Q** Tumour lung metastasis modelling was performed on the cells shown in **A** (**N**, **O**) and **I** (**P**, **Q**) to assess cell invasion and metastatic capacity in vivo and quantitative statistics were performed. Error bars represent the mean ± SD of three independent experiments (**A**, **F**, **G**, **H**, **I**, **J**), One-way repeated-measures ANOVA test.

The post-translational regulation of DACH1 by USP7 suggests that aberrant expression of USP7 could functionally promote proliferation and metastasis in CRC. Targeted knockdown of USP7 using a specific shRNAs led to a notable reduction in DACH1 protein levels in HCT8 and RKO cells. Conversely, the upregulation of DACH1 effectively reversed the decrease induced by USP7 downregulation (Figs. 5A, S5A, B). Overexpression also largely reversed the DACH1 downregulation caused by the pharmacological inhibition of USP7 (Fig. S5C, D). Conversely, specific shRNAs targeting DACH1 largely reversed the upregulation of DACH1 induced by USP7 (Fig. S5E). Colony formation assays demonstrated that the reduction in USP7 through knockdown (Fig. 5B) or pharmacological inhibition (Fig. S5F) led to a significant decrease in colony numbers in HCT8 and RKO cells. Conversely, the overexpression of DACH1 effectively counteracted this inhibitory effect. Additionally, targeting DACH1 reversed the increase in colony numbers induced by USP7 (Fig. S5G), providing further insights into the regulatory role of USP7 in CRC cell proliferation through DACH1. Additionally, transfection of HCT8 cells with specific shRNAs targeting USP7 (Fig. 5C–E) or treatment of RKO cells with the USP7 inhibitor P5091 (Fig. 5F–H) significantly inhibited tumor volume growth and weight, which was largely reversed in the context of DACH1 overexpression. Similarly, overexpression of USP7 significantly promoted tumor proliferation in SW480 cells, while targeted knockdown of DACH1 negated this promotional effect (Fig. 5I–K). Both in vivo and in vitro proliferation experiments emphasized the central role of DACH1 in USP7-mediated tumor proliferation. Exploring the impact of USP7 on invasion and migration capabilities revealed that knockdown (Fig. 5L and S5H) or pharmacological inhibition (Fig. S5I, J) of USP7 significantly reduced the invasion and migration potential of HCT8 and RKO cells, whereas overexpression of DACH1 significantly reversed this inhibitory effect. In contrast, upregulation of USP7 notably increased the invasion and migration capabilities of SW480 cells, whereas downregulation of DACH1 partially counteracted this phenomenon (Fig. 5M). Similarly, consistent findings from in vivo experiments demonstrated that the downregulation of USP7 led to a significant decrease in the number of lung metastatic nodules in HCT8 cells, whereas the upregulation of DACH1 effectively reversed this inhibitory effect (Fig. 5N, O). Moreover, the study demonstrated that the overexpression of USP7 notably increased the number of lung metastatic nodules in SW480 cells, whereas the depletion of DACH1 partially mitigated this effect (Fig. 5P, Q). Overall, these findings suggest that USP7 facilitates proliferation, invasion, and metastasis of CRC cells by stabilizing DACH1.

### GCN5-mediated acetylation of DACH1 at K680 increases its interaction with USP7

**Fig. 6:**
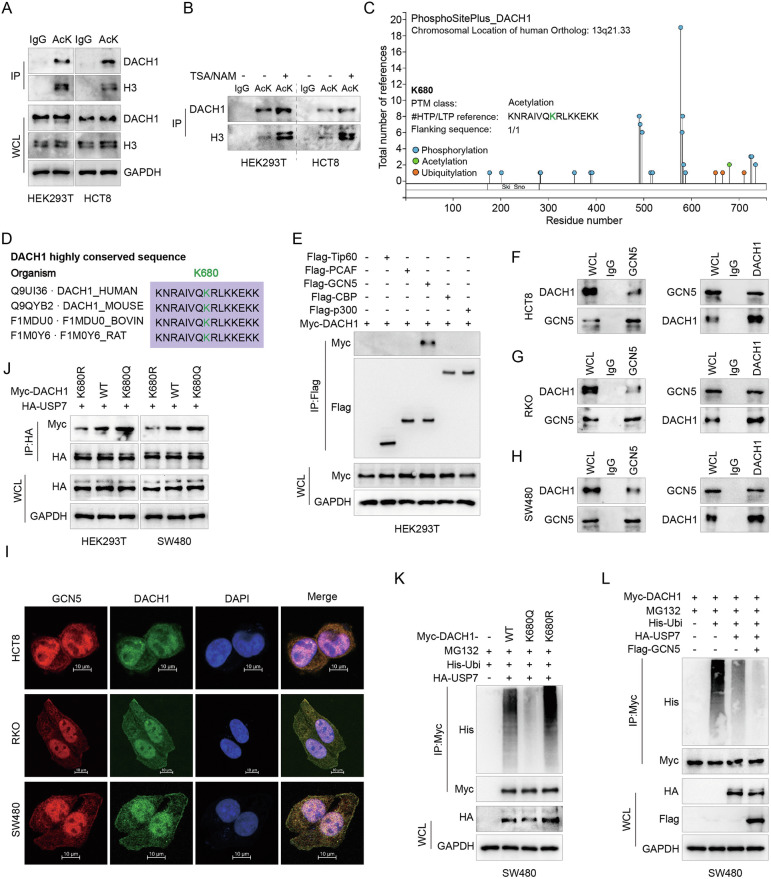
GCN5-mediated acetylation of DACH1 at K680 increases its interaction with USP7. **A** Co-IP of HEK293T and HCT8 cell lysates (treated with 5 mM NAM and 3 M TSA for 12 h prior to harvest) with acetylated lysine (AcK) antibody or IgG. Subsequent IB analysis was performed with anti-DACH1 antibody and anti-H3 antibody. **B** Protein acetylation levels were increased using NAM (5 mM, 4 h) and TSA (0.5 μM, 16 h) and acetylated DACH1 was immunoprecipitated from HEK293T and HCT8 cells with anti-AcK antibody. **C** Overview of DACH1 post-translational modification sites shown in the PhosphoSitePlus® database (https://www.phosphosite.org). **D** Amino acids adjacent to human DACH1-K680 are highly conserved across species. **E** In HEK293T cells, Myc-DACH1 was co-expressed with Flag-tagged p300, GCN5, PCAF, CBP or Tip60 acetyltransferase, respectively. Cell lysates were subjected to IP with anti-Flag antibodies, followed by IB analysis with antibodies against Myc and Flag. **F**–**H** HCT8, RKO and SW480 cells were immunoprecipitated with anti-DACH1 or anti-GCN5 and analysed. **I** Representative images of fixed HCT8, RKO and SW480 cells after immunofluorescence staining with anti-DACH1 (green) and anti-GCN5 (red) antibodies. Cell nuclei were counterstained with DAPI (blue). Scale bar is 10 μm. **J** HEK293T and SW480 cells stably expressing HA-USP7 were transfected with Myc-DACH1, Myc-DACH1 K680R or Myc-DACH1 K680Q. Cell lysates were assayed by IP with anti-HA beads. **K** SW480 cells were co-transfected with Myc-DACH1, His-Ubi, HA-USP7 and Vector-Flag or Flag-GCN5. Cell lysates were immunoprecipitated with anti-Myc beads and deubiquitinated by detection with the indicated antibodies IB. Cells were treated with 20 μM MG132 for 8 h and harvested. **L** SW480 cells were co-transfected with His-Ubi, HA-USP7 WT and Myc-DACH1 or Myc-DACH1 K680R or Myc-DACH1 K680Q. Cell lysates were immunoprecipitated with anti-Myc beads and deubiquitinated by detection with the indicated antibodies IB. Cells were treated with 20 μM MG132 for 8 h and harvested.

Acetylation, a critical lysine modification, affects various biological processes, including protein-protein interactions and enzymatic activities of numerous proteins [24, 25]. Current studies suggest that acetylation, which interacts with ubiquitination mechanisms, influences protein stability and degradation. Acetylated lysine (AcK) antibodies were used to immunoprecipitate lysates from HEK293T and HCT8 cells, followed by detection using DACH1 antibodies. These findings provide evidence that endogenous DACH1 can undergo acetylation, as demonstrated in Fig. 6A. Furthermore, the co-administration of trichostatin A (TSA) and nicotinamide (NAM), well-known deacetylase inhibitors targeting histone deacetylases (HDACs) such as HDAC I and HDAC III, as well as sirtuin-related deacetylases, resulted in increased acetylation levels of DACH1, as shown in Fig. 6B. Analysis using the PhosphoSitePlus database revealed that the K680 site in DACH1 is a highly conserved acetylation site, underscoring its potential importance in protein function (Fig. 6C, D). To investigate the acetyltransferases responsible for DACH1 acetylation, the impact of various acetyltransferases (PCAF, CBP, p300, Tip60 and GCN5) on DACH1 acetylation was evaluated. Only exogenous GCN5 interacted with exogenous DACH1 (Fig. 6E). Moreover, interactions between endogenous GCN5 and DACH1 were also observed (Fig. 6F–H). Notably, overexpression of GCN5 effectively enhanced DACH1 acetylation in SW480 and HEK293T cells (Fig. S6A). Immunofluorescence staining revealed primary nuclear colocalization of GCN5 and DACH1 (Figs. 6I and S6B). Through the expression of DACH1 WT, mutant (DACH1 K680R), and acetylation mimic (DACH1 K680Q), the necessity of DACH1 acetylation at K680 for its interaction with USP7 was investigated, showing that the K680Q mutation enhanced the interaction with USP7 (Fig. 6J). Finally, in vivo ubiquitination assays of WT and K680R mutant DACH1 (Fig. 6K), along with studies on how GCN5-mediated acetylation affects USP7-mediated deubiquitination of DACH1 (Fig. 6L), revealed that acetylation significantly reduced ubiquitination of DACH1, thereby enhancing its stability (Fig. S6C, D). These findings collectively highlight the crucial role of GCN5-mediated acetylation of DACH1 in enhancing the interaction between DACH1 and USP7, and in increasing DACH1 stability within the protein regulation network related to CRC.

### USP7 and DACH1 protein levels are positively correlated and predict poor prognosis in CRC patients

**Fig. 7:**
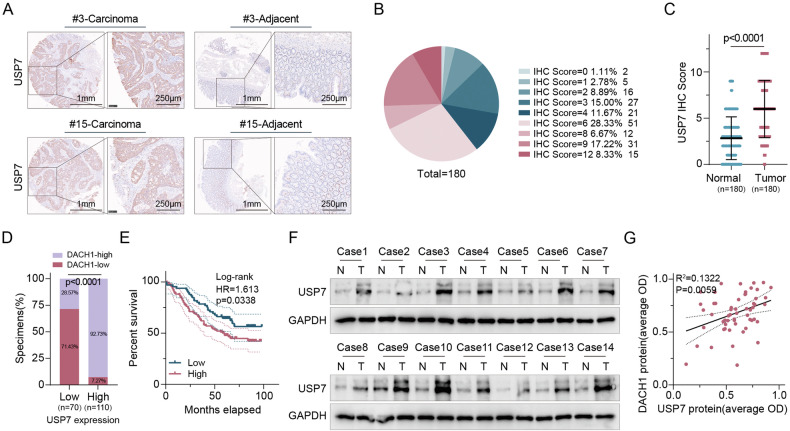
USP7 and DACH1 protein levels are positively correlated and predict poor prognosis in CRC patients. **A** Representative image of USP7 IHC staining in a human CRC sample. Scale bar, 250 µm. **B** Percentage of samples with high, medium, and low IHC staining scores. **C** USP7 IHC scores in CRC and matched paracancerous tissues. Comparison of cancer tissue with adjacent normal tissue can be performed by Wilcoxon matching test. **D** Kaplan–Meier analysis of CRC patients stratified according to high or low USP7 levels (log-rank test, P = 0.0338, n = 180). **E** Percentage of USP7 levels in 180 human CRC specimens with low or high DACH1 levels. χ2 test (two-sided). **F**, **G** IB analysis of of 14 representative CRC tumor tissues and matched neighboring tissues for USP7 and DACH1 expression (**F**). Pearson correlation between USP7 and DACH1 protein levels was performed for all 56 CRC samples (two-sided) (**G**), R2 = 0.1322, P = 0.0059.

To evaluate the relationship between USP7 and DACH1 levels in clinical CRC samples, IHC was conducted on 180 samples, comprising 70 samples with low USP7 levels and 110 samples with high USP7 levels (Fig. 7A–C). A significant correlation was observed, with 92.73% of the high USP7 samples displaying elevated DACH1 levels compared to only 28.57% in the low USP7 samples, showing a statistically significant association between increased levels of USP7 and DACH1 (P < 0.001; χ2 test) (Fig. 7D). Kaplan–Meier survival curves and log-rank tests revealed that individuals exhibiting elevated USP7 expression had a decreased overall survival rate (P = 0.0338; hazard ratio=1.613) (Fig. 7E). Furthermore, immunoblotting was performed on 56 freshly obtained CRC samples to assess USP7 and DACH1 expression levels (Fig. 7F), demonstrating a notable positive association between the protein levels of these two entities (Fig. 7G). Collectively, these findings indicate a strong correlation between the presence of USP7 and the levels of DACH1, with elevated quantities of either protein serving as potential indicators of unfavorable outcomes in individuals diagnosed with CRC.

## Discussion

In this study, the roles of DACH1 and USP7 in CRC and their post-translational regulatory mechanisms were explored, revealing their significant roles in tumor development. While previous studies have established the tumor-suppressive function of DACH1 in different cancer types, such as its ability to inhibit cyclin D1 expression to restrict renal cancer cell growth [10], or its role in antagonizing CXCL8 to suppress tumor development and enhance prognosis in lung adenocarcinoma [11], the findings of this study indicate that DACH1 may function as a tumor promoter in CRC. This apparent contradiction highlights the possibility that DACH1 operates via diverse mechanisms in various cancer types.

Moreover, this study discovered a previously unreported mechanism in which USP7 enhances the oncogenic activity of DACH1 by stabilizing it, a function not previously documented in tumor research. Previous studies on USP7 have focused on its role in regulating tumor survival and progression by stabilizing other key proteins [26]. For example, the inhibition of USP7 has been shown to induce ferroptosis in gastric cancer by targeting stearoyl-CoA desaturase [14] and inhibiting p53-independent tumor growth in triple-negative breast cancer by destabilizing FOXM1 [19]. USP7 has also been found to promote glycolysis and cell survival in non-small cell lung cancer by stabilizing and activating c-Abl [27]. USP7 accelerates p14(ARF) degradation by deubiquitinating thyroid hormone receptor-interacting protein 12 and promotes hepatocellular carcinoma progression [28]. Furthermore, USP7 was identified as a replicasome-rich SUMO deubiquitinating enzyme that is essential for DNA replication [29, 30]. By acting on SUMO and SUMOylated proteins, USP7 counteracts their ubiquitination, and inhibition or deletion of USP7 leads to accumulation of ubiquitination on SUMOylated proteins, which are removed from the replisome. All of this undoubtedly re-emphasizes the functional diversity and importance of USP7. However, the discovery of USP7’s novel role in CRC, involving direct interaction with DACH1 and regulation of its stability, presents a promising new therapeutic target for this malignancy. In addition, this study identified E3 ubiquitin ligase UHRF1 as a novel regulator of DACH1 ubiquitination. UHRF1 specifically targets DACH1 for K48-linked polyubiquitination, leading to its proteasomal degradation. However, USP7 counteracts UHRF1-mediated degradation by deubiquitinating and stabilizing DACH1, thereby promoting its oncogenic functions in CRC. These findings suggest that targeting the USP7-UHRF1 axis could offer new therapeutic strategies for modulating DACH1 stability in CRC. Comparison of the functions of DACH1 and USP7 with previous studies not only revealed their unique roles in CRC but also highlighted the potential of targeting their post-translational modifications for cancer therapy. For example, acetylation modifications, typically associated with protein stability and functional regulation, were found to enhance the interaction between DACH1 at the K680 site and USP7, which could provide clues for developing new therapeutic strategies, such as regulating DACH1’s acetylation status to affect its activity and interaction with USP7.

However, this study had some limitations. Primarily, it relies on in vitro experiments and nude mouse animal models, which may not fully replicate the complex tumor microenvironment in vivo. Furthermore, while the experimental results revealed the functional significance of the interactions between DACH1 and USP7, the universality of these interactions in clinical samples and their role in tumor heterogeneity have not been thoroughly explored. The role of UHRF1 as an E3 ligase for DACH1 also warrants further validation in clinical samples to establish its relevance in CRC progression. For future research, it is recommended to further use animal models and a broader range of clinical samples to validate and expand these findings. Given the aforementioned insights, it is imperative that future studies investigate the potential therapeutic implications of targeting DACH1 acetylation modifications or USP7 deubiquitinase activity in the context of CRC. Furthermore, a more comprehensive examination of the roles of these proteins in various cancer types and the precise mechanisms underlying their interactions are necessary to enhance our understanding of the intricate and heterogeneous nature of tumorigenesis.

## Supplementary information

A clean version of Supplemental material files

Supplemental Figure 1

Supplemental Figure 2

Supplemental Figure 3

Supplemental Figure 4

Supplemental Figure 5

Supplemental Figure 6

Table S3

Original Western Blot

## Data Availability

The datasets generated during and/or analysed during the current study are available from the corresponding author on reasonable request.
