# Supplementary material for: Synergistic regulation of DACH1 stability by acetylation and deubiquitination promotes colorectal cancer progression
Source: Cell Death Dis. 2025 May 19;16(1):400. doi: 10.1038/s41419-025-07696-9 (PMC12089419; doi:10.1038/s41419-025-07696-9)
Supplement: Supplementary file 1 — A clean version of Supplemental material files [file 41419_2025_7696_MOESM1_ESM.docx]

**Supplementary Information**

**Supplementary Materials and methods**

Cell Culture and Tissue Samples.

NCM460 was obtained from INCELL Corporation (San Antonio, Texas, USA), while CRC cell lines SW620 (CL-0225), HCT8 (CL-0098), SW480 (CL-0223), LOVO (CL-0144), RKO (CL-0196), and HEK293T (CL-0005) were obtained from Procell Life Science & Technology Co., Ltd. (Wuhan, China). These cell lines were cultured in recommended media with 10% FBS and 1% penicillin-streptomycin solution at 37°C in a 5% CO2 atmosphere. Before conducting the experiment, cell lines were verified using STR profiling. From March 2021 to December 2023, 56 pairs of CRC tissues and adjacent normal tissues were collected from patients at The First Affiliated Hospital of the University of Science and Technology of China. All participants had surgery and no prior radiotherapy or chemotherapy.

Immunoblotting (IB) and immunoprecipitation (IP) assays.

Cells were lysed in IP lysis buffer with protease inhibitors, centrifuged, and proteins were separated by SDS-PAGE for IB. Protein-protein interactions were detected by incubating lysates with antibodies and protein A/G magnetic beads, followed by washing and elution in SDS sample buffer.

qRT-PCR.

RNA was isolated using TRIzol reagent (Takara, Dalian, China) and subjected to reverse transcription with the PrimeScriptTM RT kit (Takara). Subsequently, gene expression levels were standardized to GAPDH utilizing the qRT-PCR primers detailed in Table S2.

Transwell assay.

A total of 5 x 10^4 CRC cells were placed in the top chamber of a transwell coated with Matrigel (Sigma-Aldrich). The bottom chamber contained a medium containing 15% FBS (Procell). After 20 hours at 37°C, the cells on the top surface were removed and the migrated cells were fixed and stained.

Recombinant Protein Purification.

HEK293T cells were transfected with HA-tagged USP7 or its mutant, HA-tagged USP7_C223A, and lysed in a buffer solution containing HEPES, MgCl2, KCl, glycerol, NP-40, and protease inhibitors. Cell lysates were obtained after 30 min incubation at 4°C. The cells were sonicated to release proteins and the lysate was centrifuged to remove debris. Anti-HA magnetic beads were added to the supernatant and washed with buffer to remove impurities. The HA-tagged USP7-WT/USP7_C223A proteins were isolated using elution buffer, washed, and analyzed. To isolate Myc-DACH1 from HEK293T cells, we used anti-Myc beads instead of anti-HA beads in the same steps, while keeping all other procedures consistent. To express proteins in bacteria, E. coli BL21 strain with GST and GST-USP7 plasmids was induced with 0.5 mM IPTG at 37°C for 4-6 hours. The cells were then lysed in buffer containing Tris-Cl, Triton X-100, NaCl, glycerol, DTT, and PMSF, followed by sonication. The lysate was incubated with Glutathione Sepharose 4 B at 4°C for 4 hours after centrifugation. The resin was washed multiple times with lysis buffer and PBS, and the proteins on the beads were confirmed using SDS-PAGE. They were then stored at -80°C. GST-USP7 protein was extracted using a solution containing Tris-Cl, glycerol, DTT, and reduced glutathione.

In vitro ubiquitination assay

In vitro ubiquitination assay Myc-DACH1 was expressed in HEK293T cells. After Myc-agarose pulldown, Myc-DACH1 was eluted using elution buffer containing the Myc peptide. The eluates were incubated together at 30 °C in a 25 μL reaction mixture containing 20 mм HEPES buffer (pH 7.4), 10 mм MgCl2, 1mм DTT, 1 mM ATP, 5 mм creatine phosphate, 1 U creatine kinase, 2 μм ubiquitin, 50 nм ubiquitin-like modifier activating enzyme 1 (UBE1), and 500 nм ubiquitin-conjugating enzyme E2 N (UBE2N) or ubiquitin-conjugating enzyme E2 D2 (UBE2D2) for 3 h. The reaction was stopped by adding 2 × loading buffer, boiled for 10 min, and subjected to IB.

GST pull-down Assay.

GST and GST-USP7 were purified from E. coli BL21 using GST agarose beads, followed by incubation with Myc-DACH1 from HEK293T cells at 4°C for 5 hours. Subsequently, the beads were washed three times with PBS before conducting IB analysis.

Mass spectrometry (MS) analysis.

To identify the binding proteins of DACH1, HCT8 cells were transfected with Myc-DACH1 or pcDNA3.1-vector. Lysates were immunoprecipitated with HA-beads. Beads containing affinity-bound proteins were washed three times using wash buffer (150 mм NaCl, 10 mм HEPES, pH 7.4, 0.1% NP-40), followed by elution using 1 м glycine (pH 3.0). Elutes were subjected to MS. Information on the peptides and counts for DACH1 binding proteins analyzed by IP/MS assays are provided as Table S3.

**Supplementary Figure legend**

**Supplementary Figure 1**

(A) Correlation between DACH1 mRNA expression and tumor M-stage in TCGA CRC dataset. χ2 test (two-sided).

(B) Analysis of DACH1 mRNA expression in specific subgroups of the T stage in TCGA CRC dataset. Unpaired t tests were used.

(C-F) Analysis of DACH1 IHC scores in 180 human CRC specimens in specific subgroups of T stage (C), N stage (D), M stage (E), and pathological stage(F). error bars represent the mean ± SD of three independent experiments. In C, two-sided Student’s t-test. In B, D, E, and F, One-way repeated-measures ANOVA test.

**Supplementary Figure 2**

(A) Network view of the predicted deubiquitinase (DUB) of DACH1 by UbiBrowser.

(B) SW480 cells were immunoprecipitated with anti-DACH1 or anti-USP7 antibody and analyzed.

(C) Quantification of immunofluorescence staining results and colocalization coefficients were performed using the Colocalization Finder analysis module in imageJ software.

**Supplementary Figure 3**

(A) IB and qRT-PCR analyses were performed to verify the transfection efficiency of USP7 shRNA sequences.

(B, C) Relative DACH1 mRNA expression in HCT8 (B) and RKO (C) cells transfected with control and USP7 shRNA.

(D) RKO cells transfected with two independent USP7 shRNAs were treated with or without the proteasome inhibitor MG132 (20 μM, 8h), followed by the analysis of USP7 and DACH1.

Error bars represent the mean ± SD of three independent experiments, One-way repeated-measures ANOVA test (A-D).

**Supplementary Figure 4**

(A) HEK293T cells were co-transfected with Myc-DACH1, HA-USP7, and His-Ubi WT or K48R (specifically, only K48 was mutated to Arg). His-Ubi was pulled down using Ni-NTA beads to analyze the ubiquitination linkage of DACH1.

(B) Co-IP analysis was performed to detect the interaction between DACH1 and UHRF1, MIB1, and TRIP12. Cell lysates were subjected to Myc resin, and the immunoprecipitants were subsequently blotted using the indicated antibodies.

(C) Purified Myc-DACH1 was incubated with purified Flag-UHRF1 WT in a ubiquitination assay mix, and the sample was probed for the indicated proteins.

(D, E) HA-Ubi (WT, K48R, and K63R mutants) was transfected with or without Flag-UHRF1 into HEK293T and SW480 cells. The cells were immunoprecipitated with anti-Myc and analyzed.

(F, G) HEK293T or SW480 cells were transfected with Myc-DACH1, Flag-UHRF1, and HA-tagged USP14 WT or C223A for 48 hours and analyzed by IB.

(H, I) HEK293T or SW480 cells were transfected with Myc-DACH1, Flag-UHRF1, and HA-tagged USP14 WT or C223A for 48 hours. The cells were treated with MG132 (20 µM) for 6 hours before being harvested, followed by immunoprecipitation with anti-Myc and analysis. The experiment was independently repeated three times with similar results.

**Supplementary Figure 5**

(A) IB and qRT-PCR analyses to verify the transfection efficiency of DACH1 shRNA sequences.

(B) IB analysis of HCT8 cells transduced with shRNA-Ctrl and USP7 shRNA with vector control or DACH1 plasmid.

(C-D) IB analyses of RKO and HCT8 cells under the combined intervention of pharmacological inhibition of P5091 and stable expression of DACH1.

(E) IB analysis of SW480 cells stably expressing USP7 and co-transfected with DACH1-specific shRNA.

(F) Clone formation assays for the indicated cells are shown in panels C and D.

(G) Clone formation assays for the indicated cells are shown in panel E.

(H) Transwell assay for the indicated cells (panel B).

(I, J) Transwell assays of the indicated cells are shown in panels C and D.

Error bars represent the mean ± SD of three independent experiments (B, L, M). In B, D, E, G, H, J, K, L, M, O and Q, One-way repeated-measures ANOVA test.

**Supplementary Figure 6**

(A) FLAG-GCN5 or vector was transfected into HEK293T and SW480 cells. Endogenous DACH1 was immunoprecipitated with a DACH1 antibody, followed by IB analysis with an anti-acetylated lysine antibody to detect the acetylation of DACH1 protein in the precipitates.

(B) Quantification of immunofluorescence staining results and colocalization coefficients were performed using the Colocalization Finder analysis module in imageJ software.

(C) SW480 cells were co-transfected with Myc-DACH1, HA-USP7 and Vector-Flag or Flag-GCN5 and then treated with CHX (50 μg/ml) for the indicated times, and lysates were analyzed. Graph showing the amount of DACH1 protein remaining after CHX treatment as a percentage of the initial DACH1 protein level.

(D) SW480 cells were co-transfected with USP7 and DACH1 or DACH1 K680Q and then treated with CHX (50 μg/ml) for the indicated time, and lysates were analyzed. Graph showing the amount of DACH1 protein remaining after CHX treatment as a percentage of the initial DACH1 protein level. Error bars represent the mean ± SD of three independent experiments (B and C), One-way repeated-measures ANOVA test.

**Table S1.** **The sequences of ShRNAs and SiRNAs.**

| Gene |  | Sense Sequence (5'to3') |
| --- | --- | --- |
| shControl | ShRNA | TACAAACGCTCTCATCGACAAG |
| USP7 | ShRNA1 | TGGTTCATAGTGGAGATAA |
| USP7 | ShRNA2 | AGGATTTATTCAAGATACT |
| USP7 | ShRNA3 | GTAATCCTCTTAGACATAA |
| DACH1 | ShRNA1 | AGAATAGAGCCATAGTTCA |
| DACH1 | ShRNA2 | ACGGTCTACACCAAGCTGA |
| DACH1 | ShRNA3 | TGCAACTTGCTGCTGGACA |
| UHRF1 | SiRNA1 | CGCTACGATGGCATCTACA |
| UHRF1 | SiRNA2 | AGGGTGGTGCGCAATGTCA |
| UHRF1 | SiRNA3 | GAGTAAAGTGGAGGAGACG |

**Table S2. The Primers list.**

| Gene | Forward (5'to3') | Reverse (5'to3') |
| --- | --- | --- |
| USP7 | GTCACGATGACGACCTGTCTGT | GTAATCGCTCCACCAACTGCTG |
| DACH1 | TGGAGCAGACTCTGAAAACGGG | GGTGGTTCATCTGGCTCATTGC |
| GAPDH | GACAGTCAGCCGCATCTTCT | GCGCCCAATACGACCAAATC |

**Table S3.** **Information on the peptides and counts for DACH1 binding proteins.**
