# Supplementary figures and images for: Synergistic regulation of DACH1 stability by acetylation and deubiquitination promotes colorectal cancer progression
Source: Cell Death Dis. 2025 May 19;16(1):400. doi: 10.1038/s41419-025-07696-9 (PMC12089419; doi:10.1038/s41419-025-07696-9)

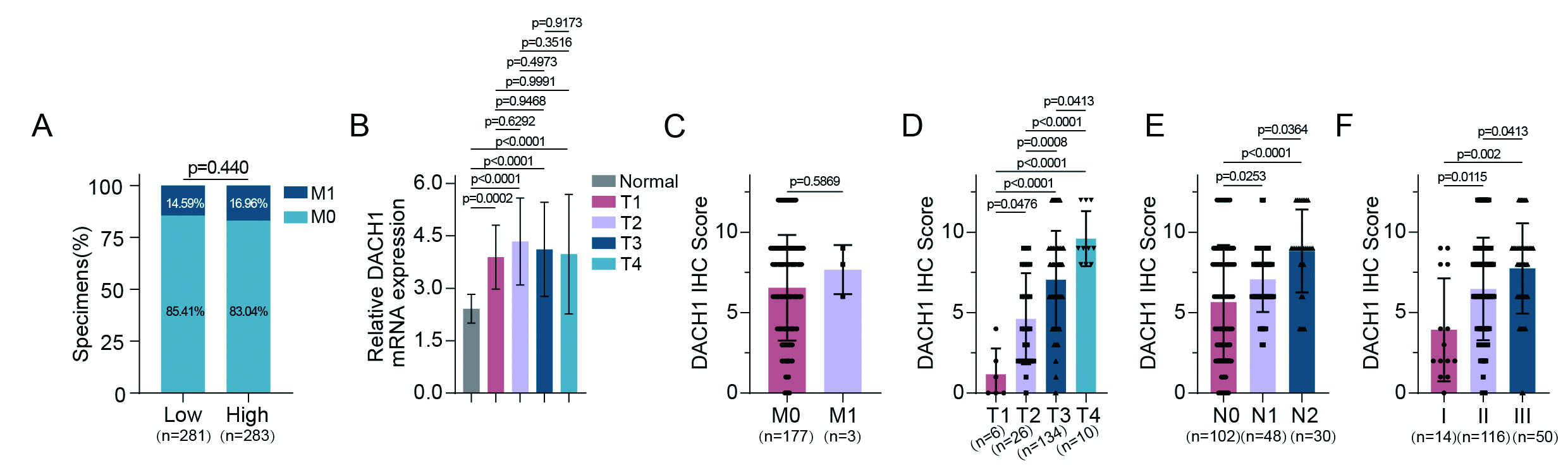

Supplement: Supplementary file 2 — Supplemental Figure 1 [file 41419_2025_7696_MOESM2_ESM.jpg]

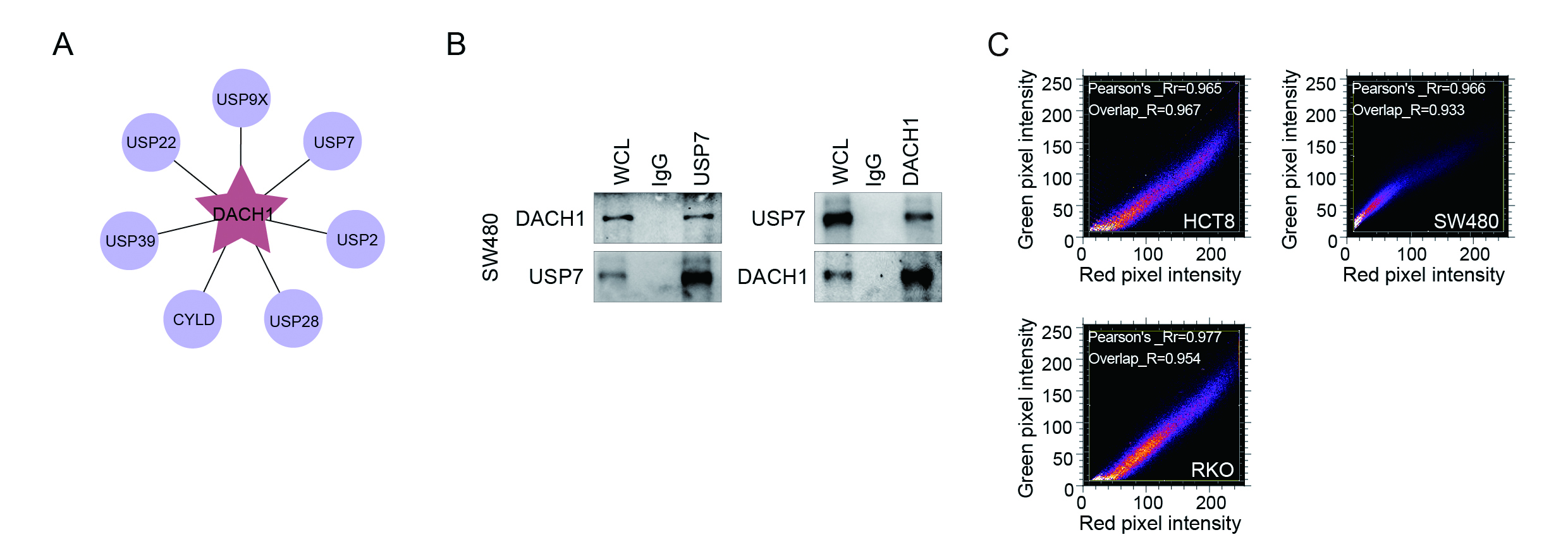

Supplement: Supplementary file 3 — Supplemental Figure 2 [file 41419_2025_7696_MOESM3_ESM.jpg]

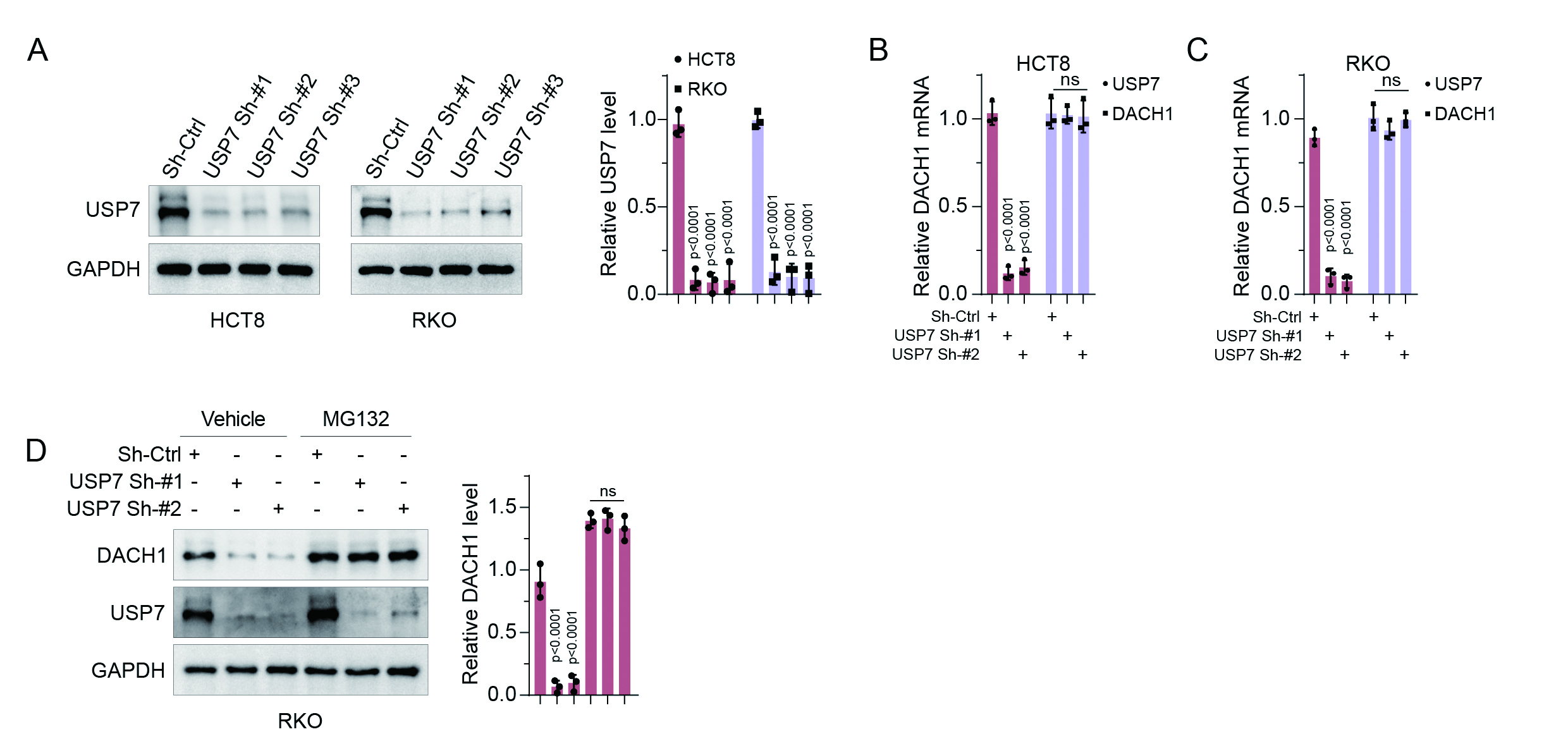

Supplement: Supplementary file 4 — Supplemental Figure 3 [file 41419_2025_7696_MOESM4_ESM.jpg]

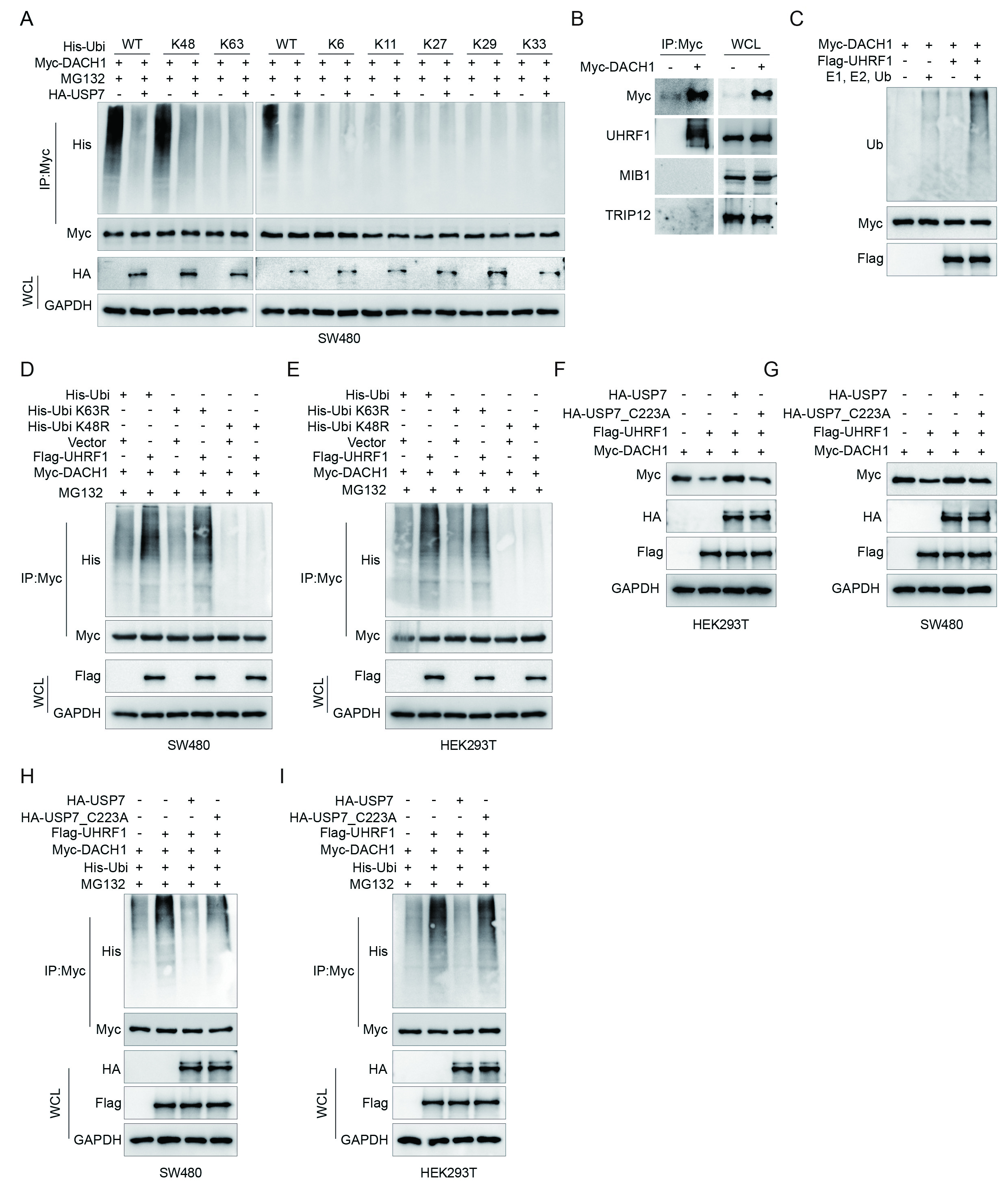

Supplement: Supplementary file 5 — Supplemental Figure 4 [file 41419_2025_7696_MOESM5_ESM.jpg]

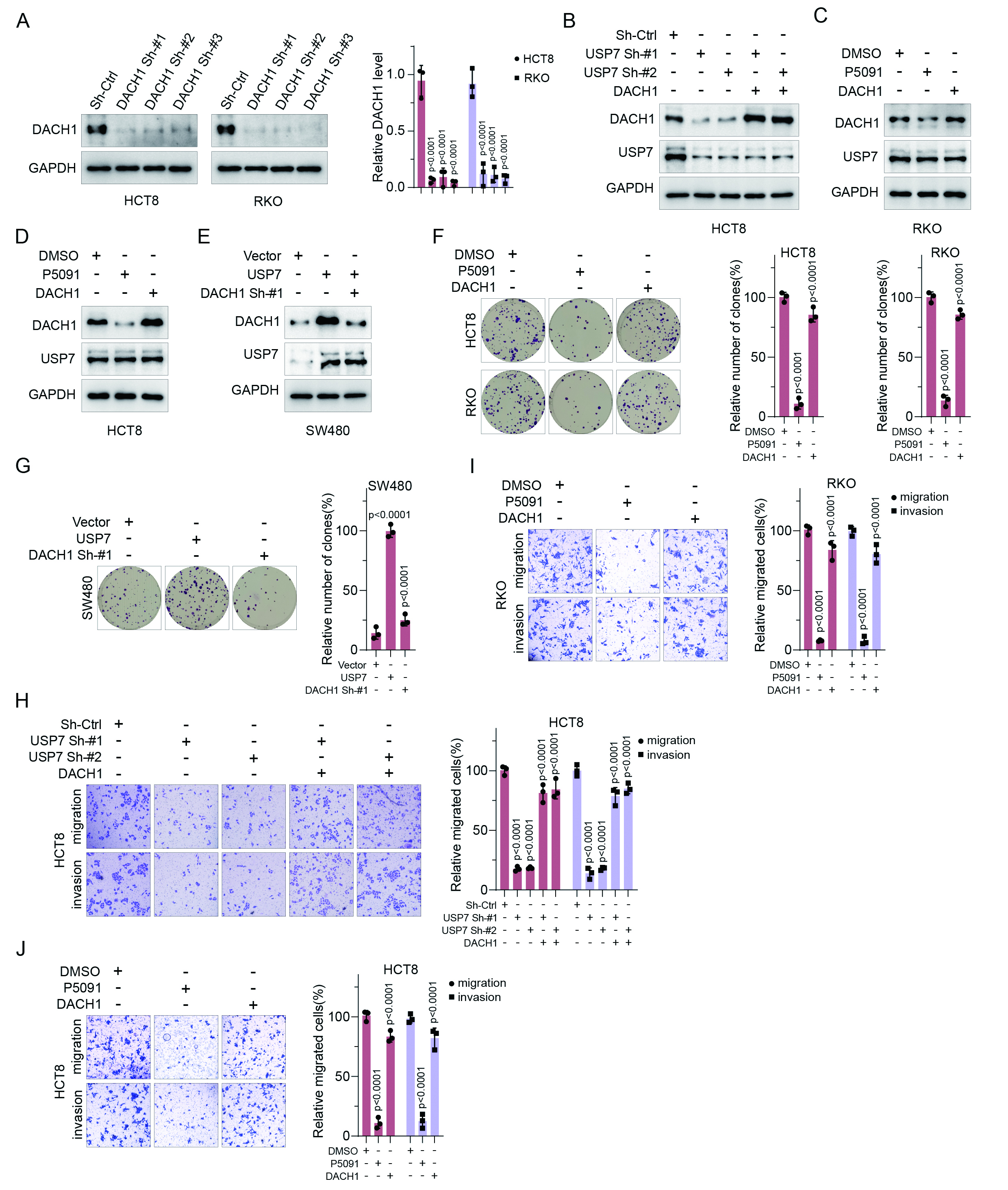

Supplement: Supplementary file 6 — Supplemental Figure 5 [file 41419_2025_7696_MOESM6_ESM.jpg]

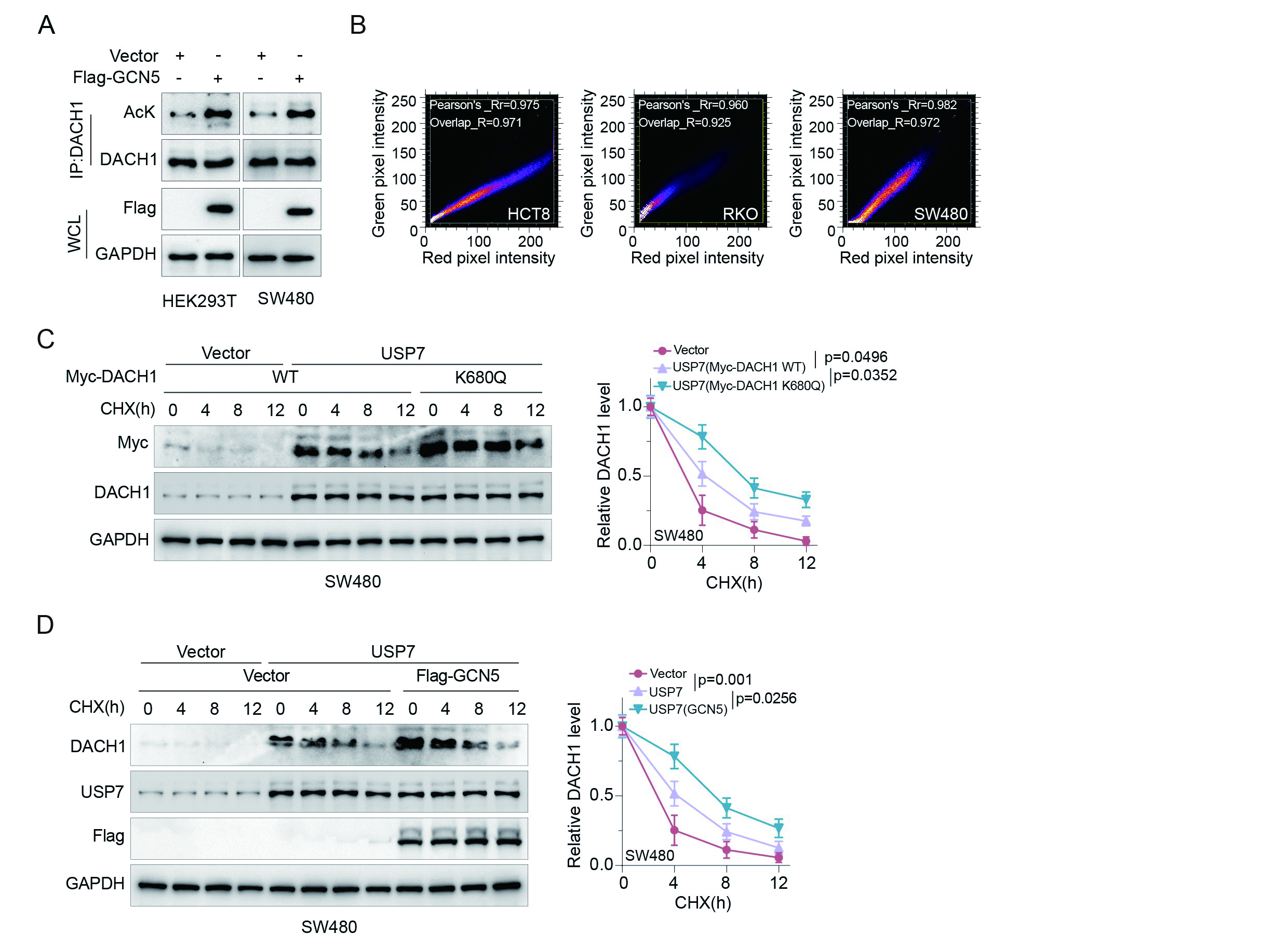

Supplement: Supplementary file 7 — Supplemental Figure 6 [file 41419_2025_7696_MOESM7_ESM.jpg]
