# Supplementary material for: Synergistic regulation of DACH1 stability by acetylation and deubiquitination promotes colorectal cancer progression
Source: Cell Death Dis. 2025 May 19;16(1):400. doi: 10.1038/s41419-025-07696-9 (PMC12089419; doi:10.1038/s41419-025-07696-9)

Figure 1N

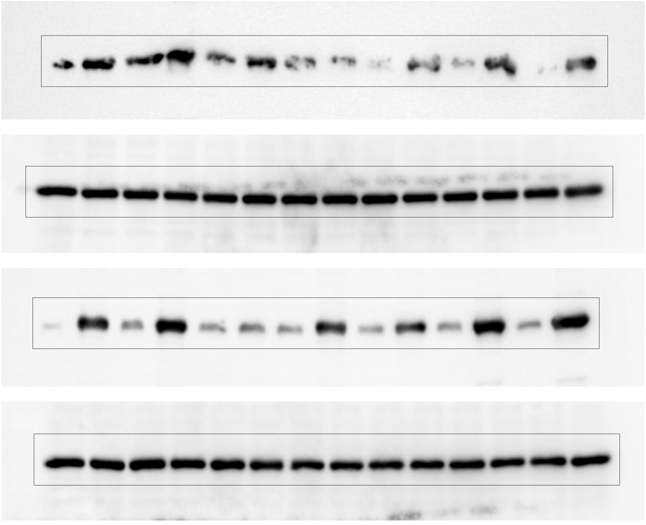

Figure 2B

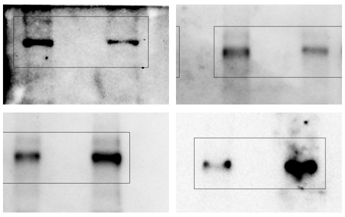

Figure 2E

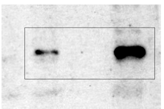

Figure 2C

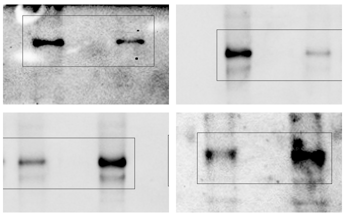

Figure S2B

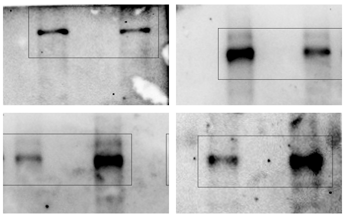

Figure 1P

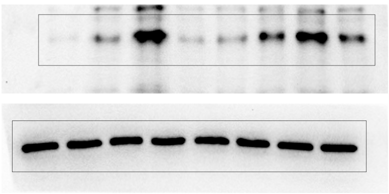

Figure 2J

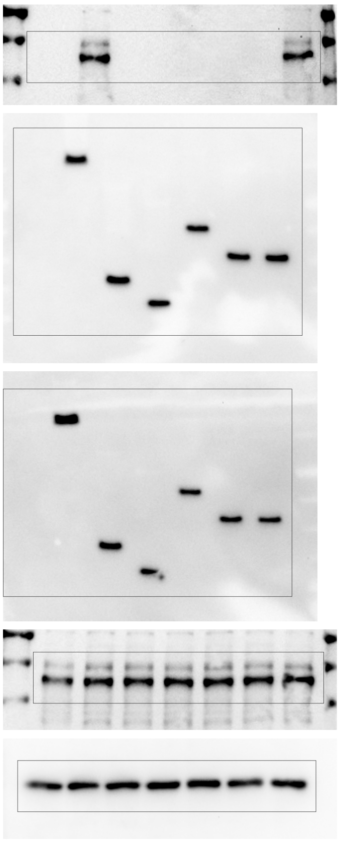

Figure 2I

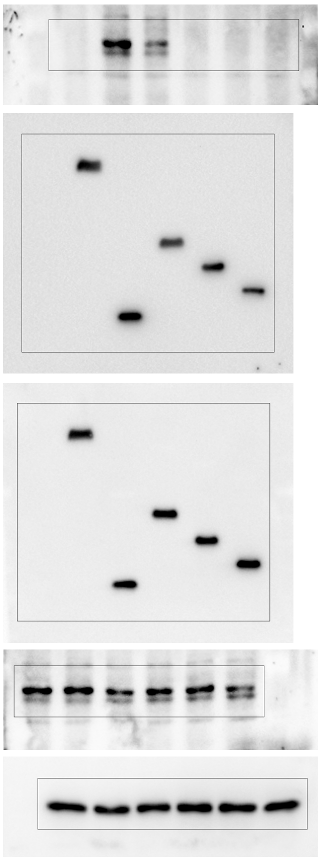

Figure 2D

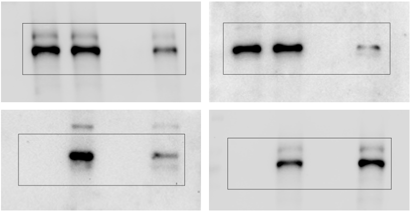

Figure 3A

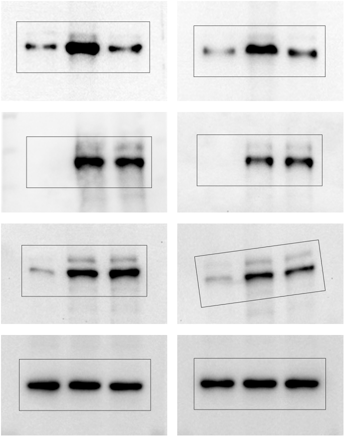

Figure S3A

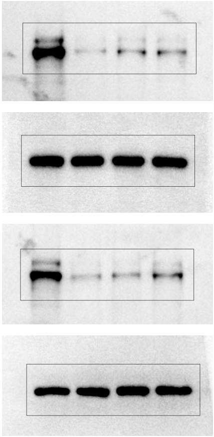

Figure 3C

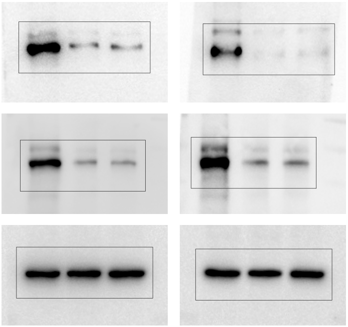

Figure 3B

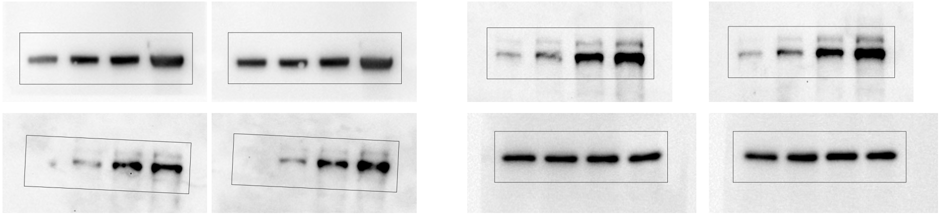

Figure 3D

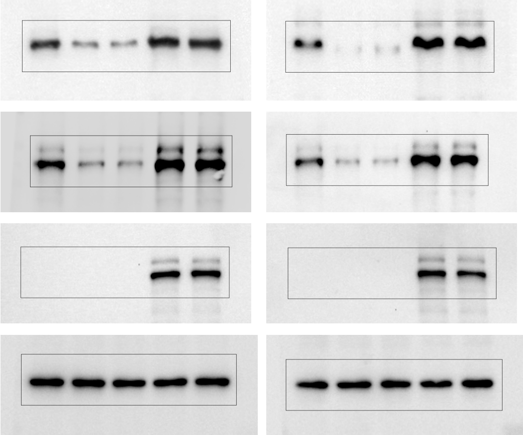

Figure 3E

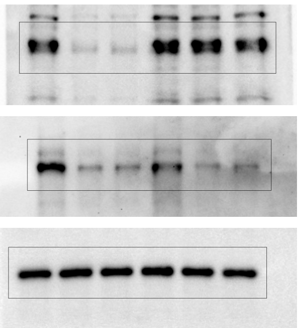

Figure S3D

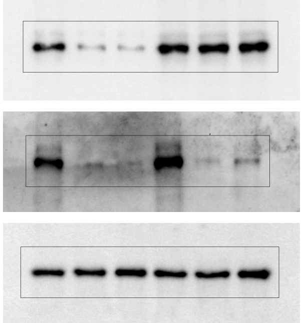

Figure 3H

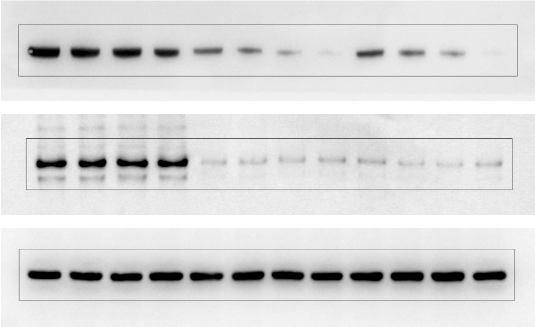

Figure 3F

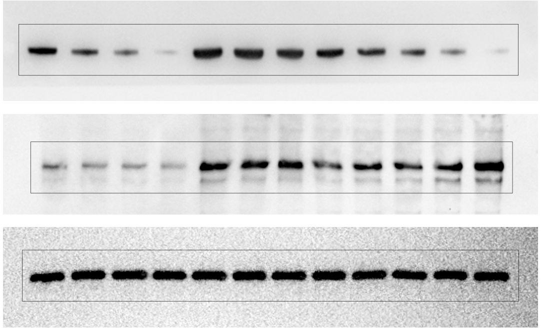

Figure 3L M

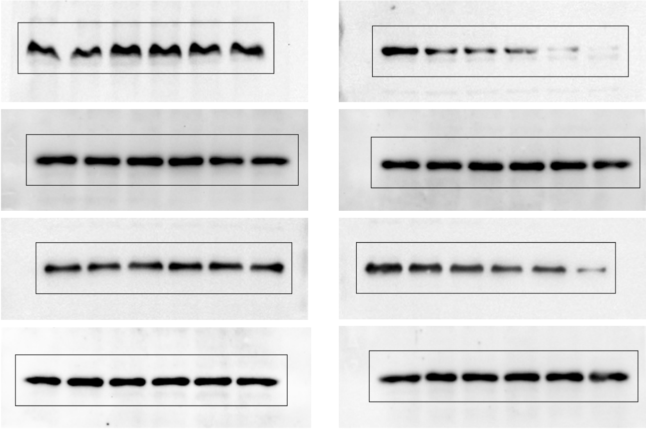

Figure 3I

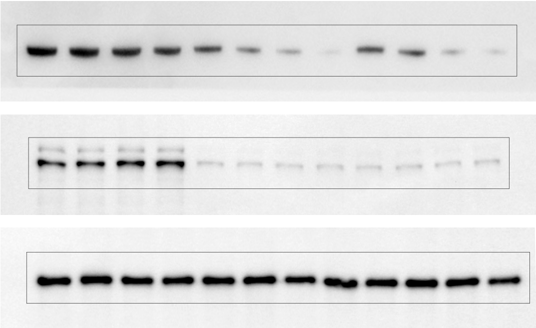

Figure 4A

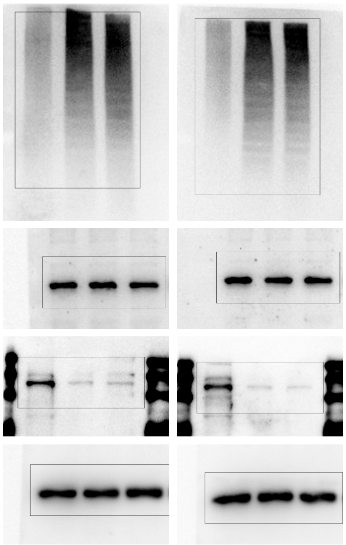

Figure 4B

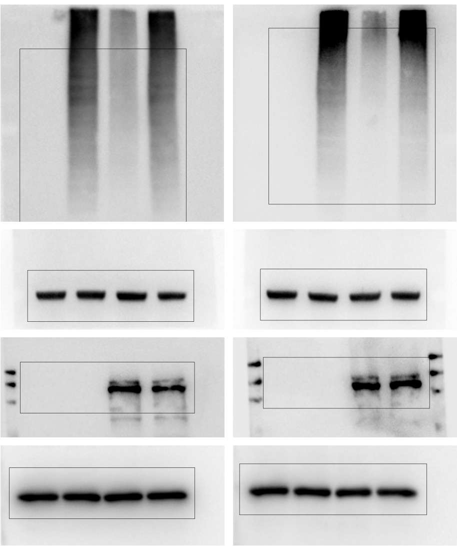

Figure 4C

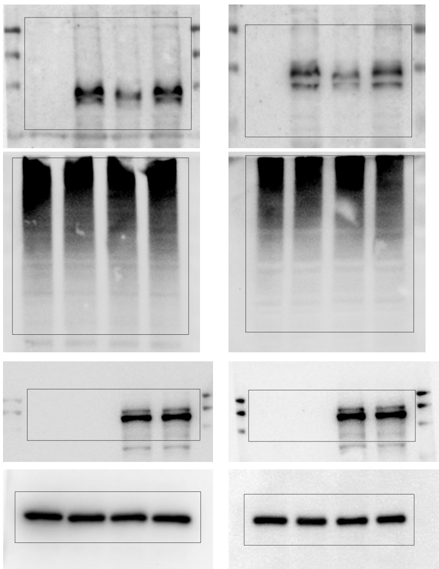

Figure 4D

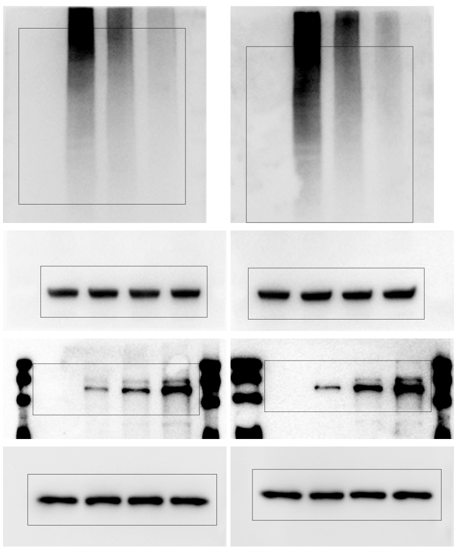

Figure 4E

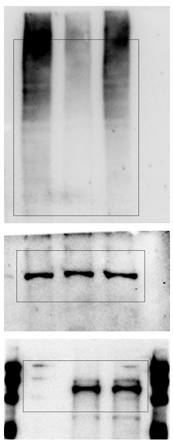

Figure 4G

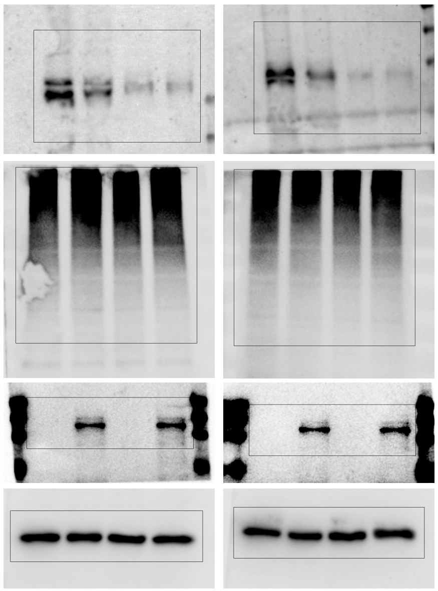

Figure 4F

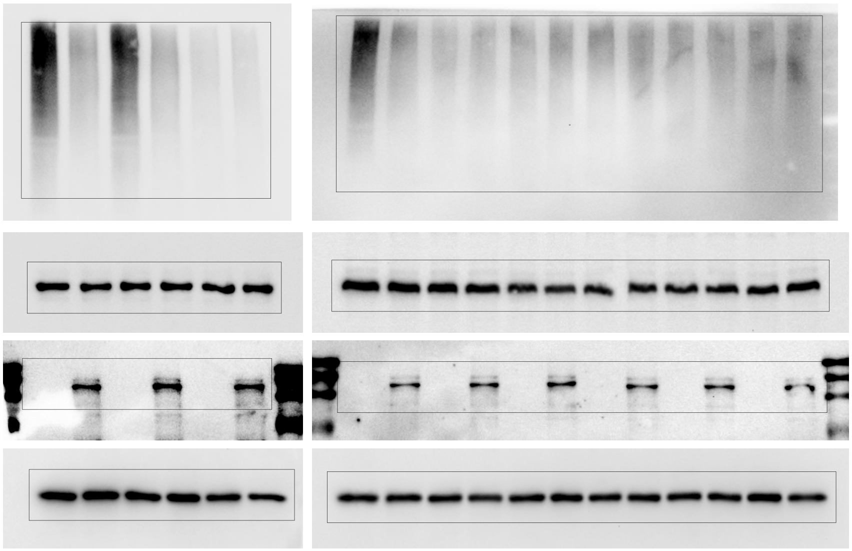

Figure 4I

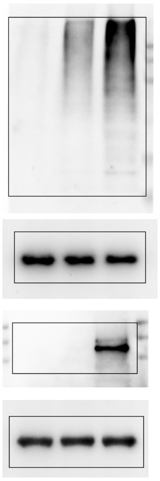

Figure 4J

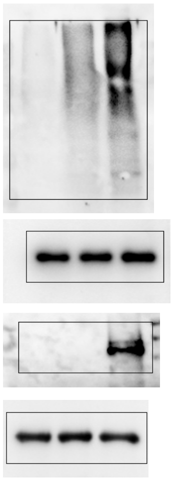

Figure 4H

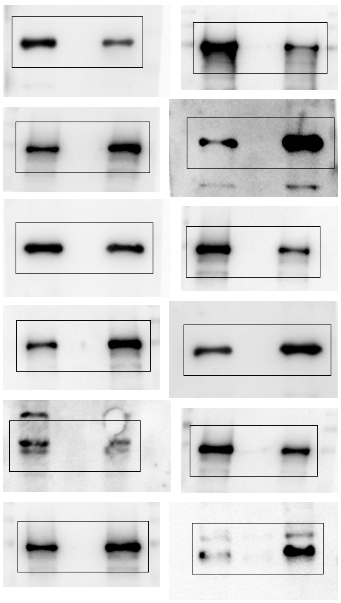

Figure 4K

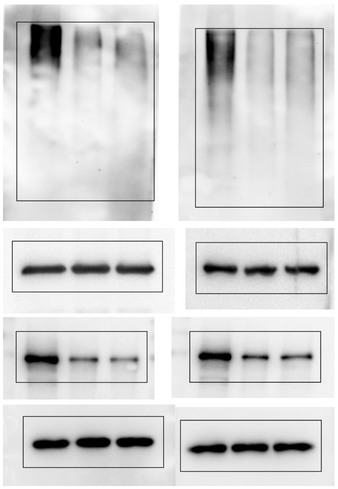

Figure S4B

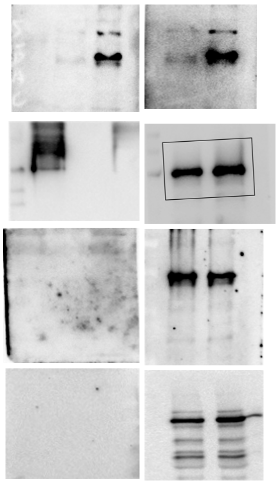

Figure S4D

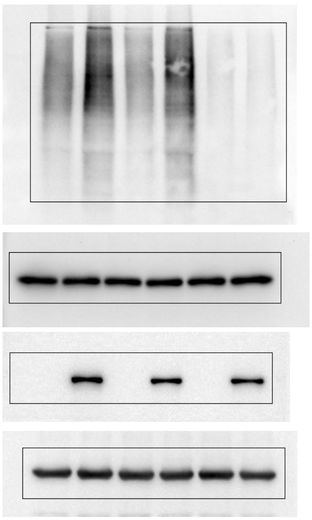

Figure S4C

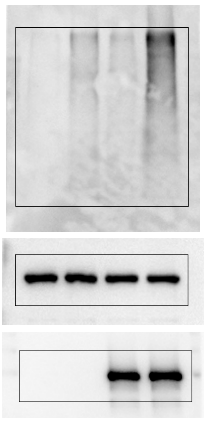

Figure S4F

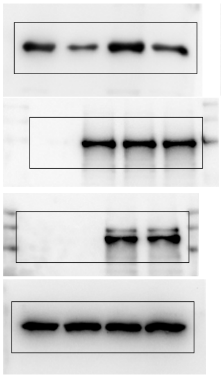

Figure S4G

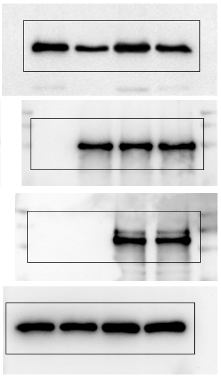

Figure S4A

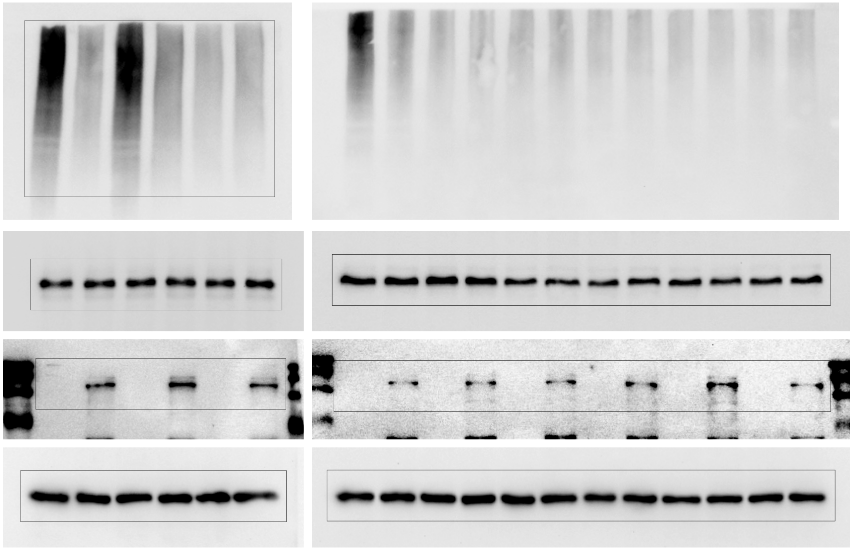

Figure S4F

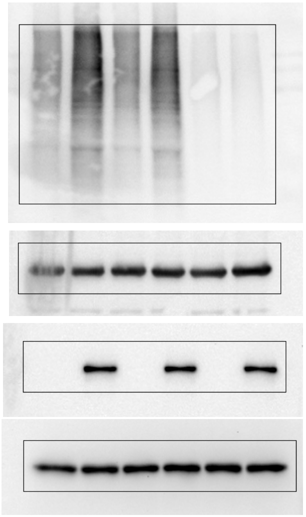

Figure S4H

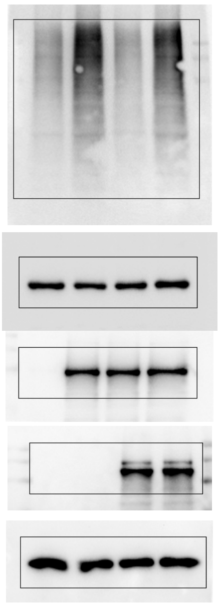

Figure S4I

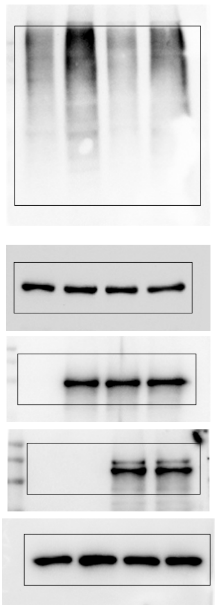

Figure 5 A

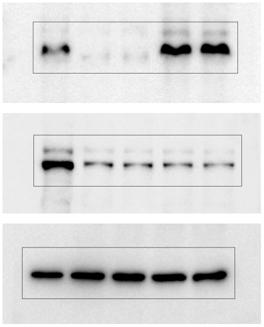

Figure S5 A

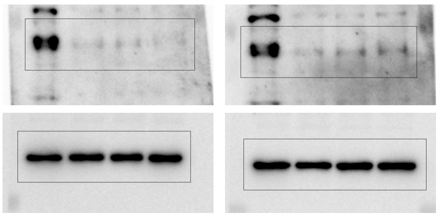

Figure S5 D

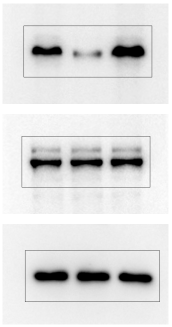

Figure S5 E

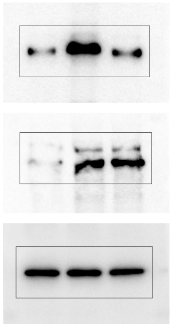

Figure S5 B

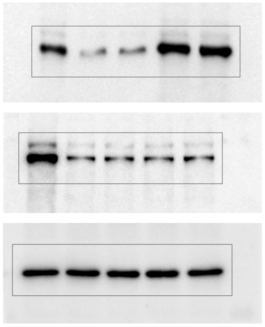

Figure S5 C

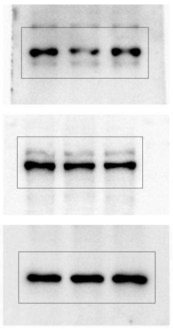

Figure 6 A

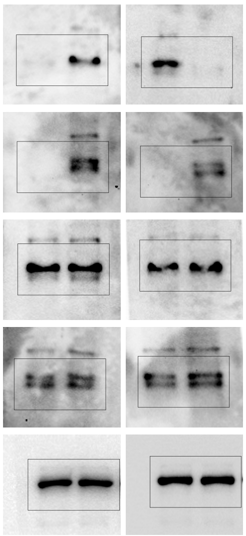

Figure 6 B

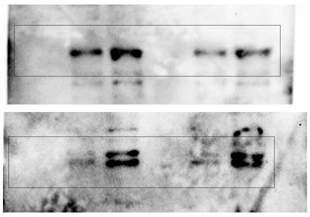

Figure 6 E

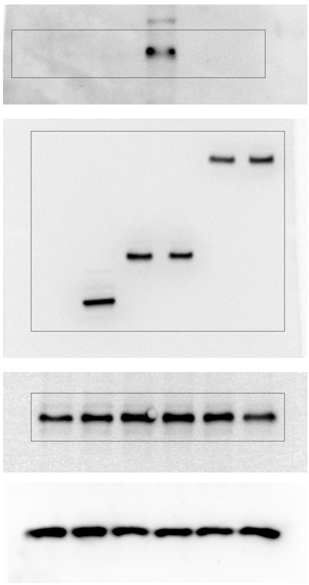

Figure 6 F

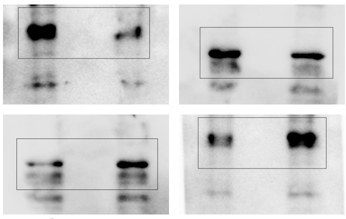

Figure 6 G

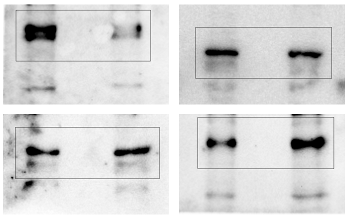

Figure 6 H

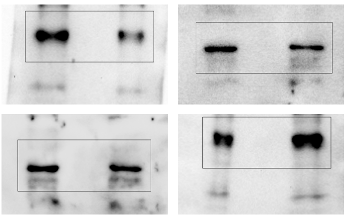

Figure 6 J

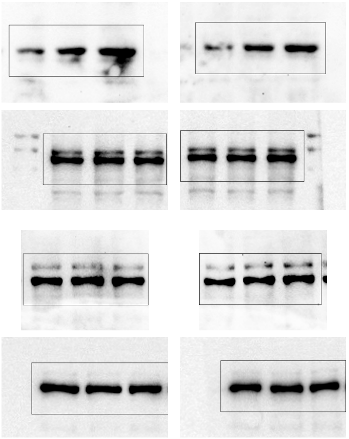

Figure 6 K

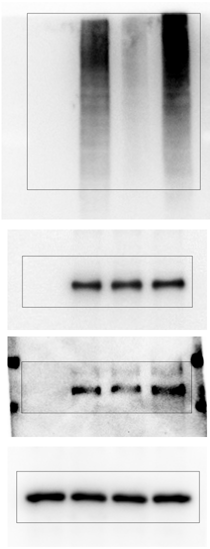

Figure 6 L

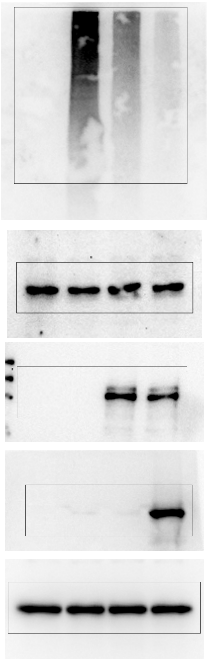

Figure S6 A

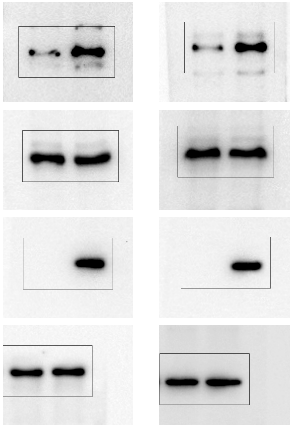

Figure S6 C

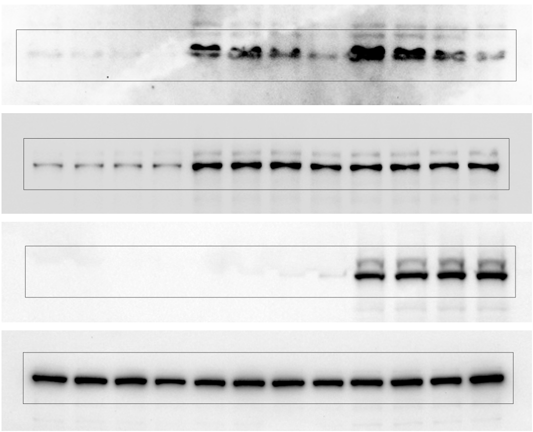

Figure 7F

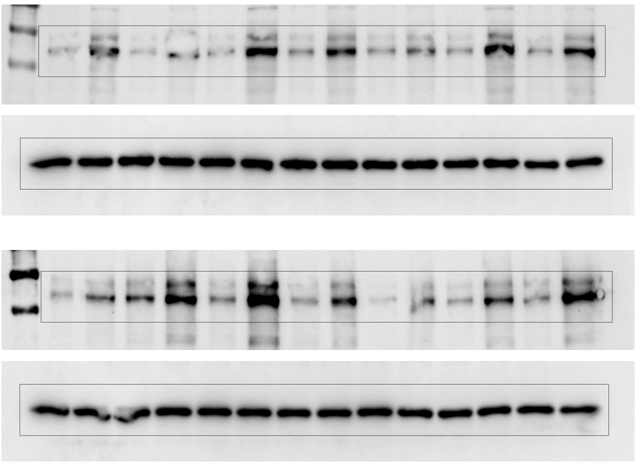

Figure S6 D

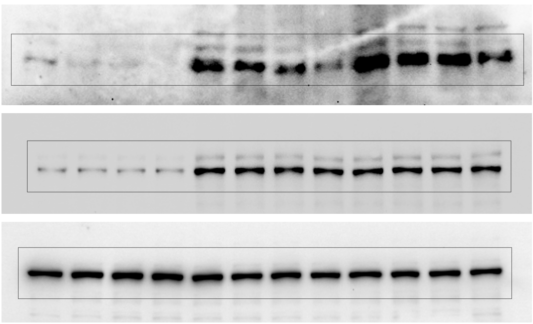

Supplement: Supplementary file 9 — Original Western Blot [file 41419_2025_7696_MOESM9_ESM.pdf]
